# Long non-coding RNAs in glioblastoma: from molecular drivers to therapeutic targets

**DOI:** 10.3389/fimmu.2026.1887815

**Published:** 2026-08-07

**Authors:** Noha M. Elemam, Jana H. Sweillam, Youssef El-Sherif, Yomna A. Youssef, Habiba M. Ismail, Reem A. Assal, Rana A. Youness

**Affiliations:** 1 Clinical Sciences Department, College of Medicine, University of Sharjah, Sharjah, United Arab Emirates; 2 Research Institute for Medical and Health Sciences, University of Sharjah, Sharjah, United Arab Emirates; 3 M.Sc. Molecular Medicine Program, Faculty of Medicine, Friedrich Schiller University Jena, Jena, Germany; 4 Department of Medical Biotechnology, College of Biotechnology, Misr University for Science and Technology, Giza, Egypt; 5 Molecular Genetics Research Team (MGRT), Molecular Biology and Biochemistry Department, Faculty of Biotechnology, German International University, New Administrative Capital, Egypt; 6 Physiology Department, Faculty of Physical Therapy, German International University, New Administrative Capital, Egypt; 7 Department of Pharmacology and Toxicology, Faculty of Pharmacy, Heliopolis University for Sustainable Development (HU), Cairo, Egypt

**Keywords:** biomarkers, drug delivery, epigenetics, glioblastoma, lncRNAs, oncogenic, therapeutic targets

## Abstract

Glioblastoma (GBM) is the most aggressive primary malignant brain tumor in adults and remains associated with poor clinical outcomes despite advances in surgical resection, radiotherapy, and temozolomide-based chemotherapy. Its remarkable molecular heterogeneity, highly immunosuppressive tumor microenvironment, and intrinsic therapeutic resistance continue to limit the effectiveness of current treatment strategies. Long non-coding RNAs (lncRNAs), transcripts exceeding 200 nucleotides in length that lack protein-coding capacity, have emerged as critical regulators of GBM biology through transcriptional, post-transcriptional, and epigenetic mechanisms. Acting as competing endogenous RNAs, chromatin modifiers, molecular scaffolds, and regulators of RNA-binding proteins, lncRNAs orchestrate key oncogenic pathways controlling proliferation, invasion, angiogenesis, stemness, metabolic reprogramming, immune evasion, and resistance to chemotherapy and radiotherapy. This review provides a comprehensive overview of lncRNA biogenesis, classification, and mechanisms of action, followed by an updated synthesis of oncogenic and tumor-suppressive lncRNAs implicated in GBM progression. Particular emphasis is placed on emerging evidence demonstrating how immunophenotype-related lncRNAs regulate immune cell infiltration, immune checkpoint signaling, and the immunosuppressive glioblastoma microenvironment, highlighting their potential to improve patient stratification and guide immunotherapeutic approaches. We also discuss the growing clinical utility of lncRNAs as diagnostic, prognostic, and predictive biomarkers, including circulating lncRNAs and lncRNA-based molecular signatures. Finally, we examine current and emerging therapeutic strategies targeting lncRNAs, including nanoparticle-mediated delivery systems designed to overcome the blood-brain barrier. Advances in single-cell and spatial transcriptomic technologies are further expanding our understanding of lncRNA-mediated regulatory networks and intratumoral heterogeneity, supporting the development of precision medicine strategies. Collectively, lncRNAs represent promising biomarkers and therapeutic targets with significant potential to improve the diagnosis, prognosis, and treatment of glioblastoma.

## 1 Introduction to glioblastoma

Primary central nervous system (CNS) tumors comprise a wide variety of neoplasms that originate from the brain, meninges, spinal cord, or cranial nerves (1). There are over a hundred diverse subtypes of CNS tumors, both benign and malignant, whose pathogenesis is influenced by factors such as age, gender, race, environmental factors, and genetics (2, 3). Among malignant CNS tumors, glioblastoma (GBM) is ranked the most aggressive and the most prevalent, accounting for 45.2% of primary malignant CNS tumors (4). Globally, annual incidence rates of GBM range from 0.59 to 5 per 100,000 persons and are increasing (5–8). In low- and middle-income countries, over 940,000 cases of CNS tumors are diagnosed annually (9). This is assumed to be lower than the actual prevalence due to underdiagnosis of GBM in these countries, which is mainly associated with poor screening and lack of awareness (10, 11). Unfortunately, the delay in diagnosis worsens the already-poor prognosis of GBM. Despite the remarkable advancement in cancer research throughout the past decades, survival outcomes of GBM range between 6 and 21 months (12). The currently available treatment options are limited to surgical removal of the tumor and a combination of chemotherapy and radiotherapy (13). However, GBM is considered one of the most treatment-resistant malignancies, and successful treatment does not prevent the tumor’s recurrence.

GBM is classified as a CNS WHO grade 4 diffuse glioma according to the fifth edition of the WHO Classification of Tumors of the Central Nervous System (WHO CNS5), representing the most aggressive primary brain tumor in adults and one of the deadliest malignancies of the central nervous system (14). Despite advances in surgery, radiotherapy, and chemotherapy, the prognosis remains poor because of the tumor’s highly infiltrative nature, remarkable biological heterogeneity, and resistance to conventional therapies (15, 16).

One of the defining characteristics of GBM is its extensive intertumoral and intratumoral heterogeneity, encompassing histopathological, molecular, cellular, and clinical features. This heterogeneity contributes to marked differences in tumor progression, therapeutic response, recurrence, and overall patient survival, highlighting the importance of integrating molecular biomarkers into conventional histopathological diagnosis (16, 17).

The current WHO CNS5 classification incorporates both histopathological and molecular characteristics to improve diagnostic accuracy and prognostic stratification (14). Histopathologically, GBM is characterized by diffuse astrocytic growth with microvascular proliferation and/or necrosis. At the molecular level, adult-type diffuse gliomas are classified into three major entities: astrocytoma, IDH-mutant; oligodendroglioma, IDH-mutant and 1p/19q-codeleted; and GBM, IDH-wildtype (14). In addition to IDH mutation status, several molecular alterations—including EGFR amplification, TERT promoter mutations, chromosome 7 gain/chromosome 10 loss (+7/−10), ATRX loss, TP53 mutations, and MGMT promoter methylation—play important roles in tumor classification, prognosis, and therapeutic response (18).

Historically, the 2016 WHO classification first established the distinction between IDH-wildtype and IDH-mutant glioblastomas, recognizing IDH mutation status as a major prognostic marker (19). Although this distinction remains biologically relevant, current evidence indicates that glioma classification extends beyond IDH status alone and requires integration of multiple molecular and clinicopathological features (14). GBM, IDH-wildtype is the predominant subtype, accounting for approximately 90% of cases, and is characterized by frequent EGFR amplification, TERT promoter mutations, chromosome 10 loss, rapid tumor progression, and poor clinical outcomes (18, 20).

Conversely, IDH-mutant astrocytic tumors are generally associated with younger age at diagnosis and a more favorable prognosis than IDH-wildtype GBMs owing to their distinct molecular profile, including frequent IDH1/IDH2 mutations, ATRX loss, and TP53 mutations (18). However, the presence of an IDH mutation should not be interpreted as indicating indolent disease. High-grade IDH-mutant astrocytomas, particularly CNS WHO grade 4 tumors, exhibit aggressive biological behavior characterized by diffuse infiltration, high proliferative capacity, therapeutic resistance, and inevitable recurrence (21). Additional molecular alterations acquired during tumor evolution, particularly homozygous deletion of CDKN2A/B, can further promote malignant progression and significantly worsen prognosis despite the presence of an IDH mutation (21). Therefore, prognosis depends on the combined influence of molecular, histopathological, and clinical factors rather than on IDH mutation status alone (21). The principal clinicopathological and molecular differences between glioblastoma, IDH-wildtype, and astrocytoma, IDH-mutant (CNS WHO grade 4) are summarized in **Table 1** (21).

**Table 1:** Comparison of glioblastoma, IDH-wildtype and astrocytoma, IDH-mutant (CNS WHO grade 4).

Feature	GBM, IDH-wildtype	Astrocytoma, IDH-Mutant (CNS WHO Grade 4)
WHO CNS5 Classification	Adult-type diffuse glioma	Adult-type diffuse glioma
Typical Origin	Usually arises *de novo* (primary)	Frequently evolves from lower-grade IDH-mutant astrocytoma
Incidence	~ 90% of GBMs	Less common
Typical Age	Older adults	Younger adults
Major Molecular Alterations	EGFR amplification, TERT promoter mutation, +7/−10 signature	IDH1/IDH2 mutation, TP53 mutation, ATRX loss, CDKN2A/B homozygous deletion (grade 4)
Histopathological Features	Diffuse astrocytic tumor with necrosis and/or microvascular proliferation	Diffuse astrocytic tumor; grade 4 tumors may also show necrosis and microvascular proliferation
IDH1 R132H Immunostaining	Negative	Usually positive
Biological Behavior	Highly aggressive, rapidly progressive	Better overall survival but grade 4 tumors remain highly aggressive, infiltrative, and prone to recurrence
Prognosis	Poor	Generally better than IDH-wildtype GBM but still poor in grade 4 disease

Current diagnostic evaluation of suspected GBM begins with contrast-enhanced magnetic resonance imaging (MRI), followed by histopathological examination and molecular profiling to establish the diagnosis and guide treatment decisions (14, 22). Among the clinically relevant biomarkers, MGMT promoter methylation predicts improved responsiveness to the alkylating agent temozolomide and remains an important predictive marker in routine clinical practice (23). The growing recognition of GBM as a molecularly heterogeneous disease has stimulated the search for novel biomarkers to improve diagnosis, prognostic stratification, and therapeutic targeting (14, 16). In this context, long non-coding RNAs (lncRNAs) have emerged as promising regulators of glioma biology due to their roles in tumor proliferation, invasion, stemness, angiogenesis, immune modulation, and treatment resistance (24, 25).

In addition to its extensive genetic and epigenetic heterogeneity, GBM is characterized by a profoundly immunosuppressive tumor microenvironment that enables efficient immune evasion and contributes to disease progression. GBM cells employ multiple mechanisms to escape immune surveillance, including the recruitment of immunosuppressive immune populations, secretion of inhibitory cytokines, induction of T-cell exhaustion, and upregulation of immune checkpoint molecules, collectively resulting in impaired anti-tumor immune responses (26, 27). Moreover, the blood-brain barrier (BBB) represents a major obstacle to effective therapy by restricting the penetration of many therapeutic agents, including several immunotherapeutic compounds, into the tumor site (28). Although immune checkpoint inhibitors have revolutionized the treatment of several solid malignancies, their efficacy in GBM has remained limited, with clinical trials failing to demonstrate substantial improvements in overall survival (29). These challenges underscore the urgent need to identify novel molecular regulators of the GBM immune microenvironment that may serve as predictive biomarkers and therapeutic targets to enhance anti-tumor immunity.

While noncoding RNAs, especially long non-coding RNAs, are well recognized as key regulators of GBM development and progression, their contribution to the establishment and maintenance of the immunosuppressive tumor microenvironment remains underexplored. This review addresses the pivotal question of how lncRNAs regulate oncogenic pathways in GBM, govern immune cell function, coordinate immune signaling networks, influence treatment responsiveness in glioblastoma, and whether their therapeutic modulation could improve anti-tumor immune activity. Through an integrated analysis of molecular mechanisms, immune regulation, biomarker applications, and therapeutic opportunities, this review highlights lncRNAs as important mediators connecting glioblastoma biology and emerging immunotherapeutic strategies.

## 2 Non-coding RNAs

Noncoding RNAs (ncRNAs), though they do not encode proteins, make up the majority of RNA in the human genome and play fundamental roles in both normal physiology and cancer, including angiogenesis, proliferation, differentiation, and metastasis (30–34). Depending on the ncRNA, dysregulation can drive either tumor suppression or oncogenesis, with implications for diagnosis, prognosis, and therapy (35).

NcRNAs are classified by size. MicroRNAs (miRNAs) are short (~18–22 nucleotide) ncRNAs, first identified in 2000 with let-7 (36, 37); over 2,500 have since been discovered, and altered miRNA expression is common across cancers. In GBM, miR-21 is an oncogenic miRNA that promotes tumor progression by inhibiting p53 and activating EGFR and Cyclin D1, while miR-1 acts as a tumor suppressor and is typically downregulated (38, 39).

Circular RNAs (circRNAs) are closed-loop, single-stranded ncRNAs that often function as miRNA “sponges” via miRNA response elements (40, 41). For example, circ_0021350 sequesters miR-1207-3p in GBM, promoting proliferation and metastasis by regulating PIK3R3 (42, 43).

Long noncoding RNAs (lncRNAs) are especially abundant in the human brain, more so than in any other organ studied, reflecting the nervous system’s complexity (44–46). LncRNAs regulate gene expression at both transcriptional and post-transcriptional levels, shaping neuronal function in health and disease, and have emerged over the past decade as key regulators of tumor initiation, progression, and metastasis (47).

## 3 Long non-coding RNAs

**Figure 1:**
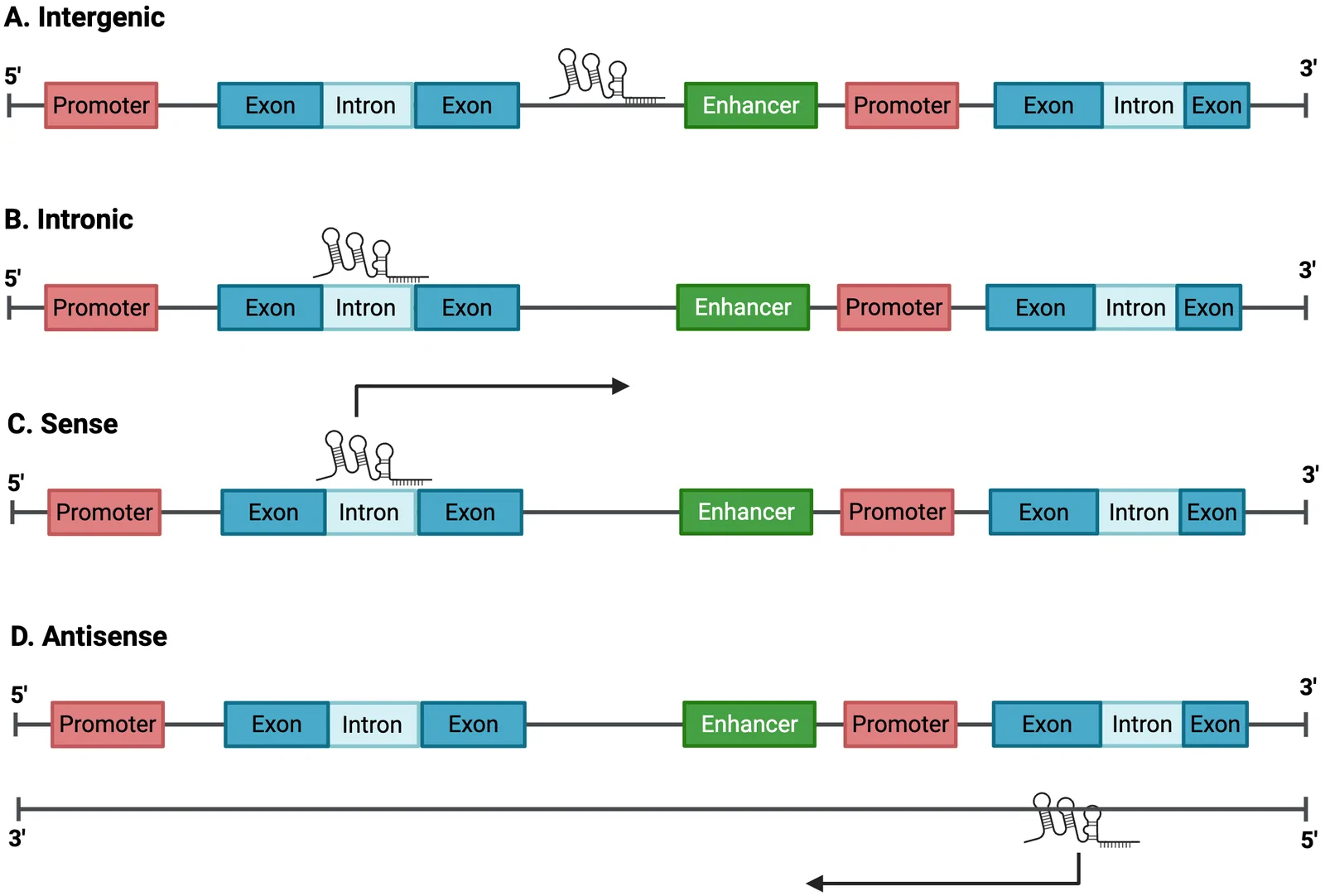
Subclasses of lncRNA. **(A)** Intergenic lncRNAs are located between two coding genes, whereas **(B)** lncRNAs transcribed from within the intronic region of a protein-coding gene are called intronic lncRNAs. **(C)** Sense lncRNAs are transcribed from the same strand and in the same direction as protein-coding genes. **(D)** Antisense lncRNAs are transcribed from the opposite strand.

LncRNAs are the most diverse class of ncRNAs, ranging from several hundred to thousands of nucleotides in length, with GENCODE (v47) reporting over 35,000 lncRNA genes and other estimates exceeding 120,000 (48, 49). They are classified by their position relative to protein-coding genes, intergenic, intronic, sense, and antisense (**Figure 1**), a distinction that also correlates with regulatory function, as well as by functional and targeting mechanisms, discussed later (50).

Naming conventions established by the HUGO Gene Nomenclature Committee (HGNC), GENCODE, and others aim to ensure individuality, descriptiveness, and uniformity across lncRNA gene names (51, 52). LncRNAs are preferentially named after their function or phenotype (e.g., HOTAIR) or genomic location (e.g., MALAT-1). Given their rapid expansion and diversity, standardized nomenclature is increasingly important for consistency and cross-study comparability.

### 3.1 Biogenesis of lncRNAs

LncRNAs (>200 nucleotides) undergo a multi-stage biogenesis beginning with transcription by RNA polymerase II, the same enzyme used for mRNAs (53). Unlike mRNAs, they arise from diverse genomic regions, giving rise to five classes based on genetic origin: sense (overlapping exons on the same strand), antisense (overlapping but transcribed in the opposite direction), bidirectional (near a promoter, transcribed in reverse), intronic, and intergenic lncRNAs (54). Functionally, lncRNAs are commonly classified into four categories based on their mechanism of action: signal (indicating specific transcriptional responses), decoy (sequestering regulatory proteins or miRNAs), guide (directing ribonucleoprotein complexes to genomic loci), and scaffold (serving as structural platforms for assembling functional complexes). They are also categorized by subcellular location (nuclear, cytoplasmic, chromatin-associated, mitochondrial), each contributing distinctly to gene expression and cellular activity (54).

**Figure 2:**
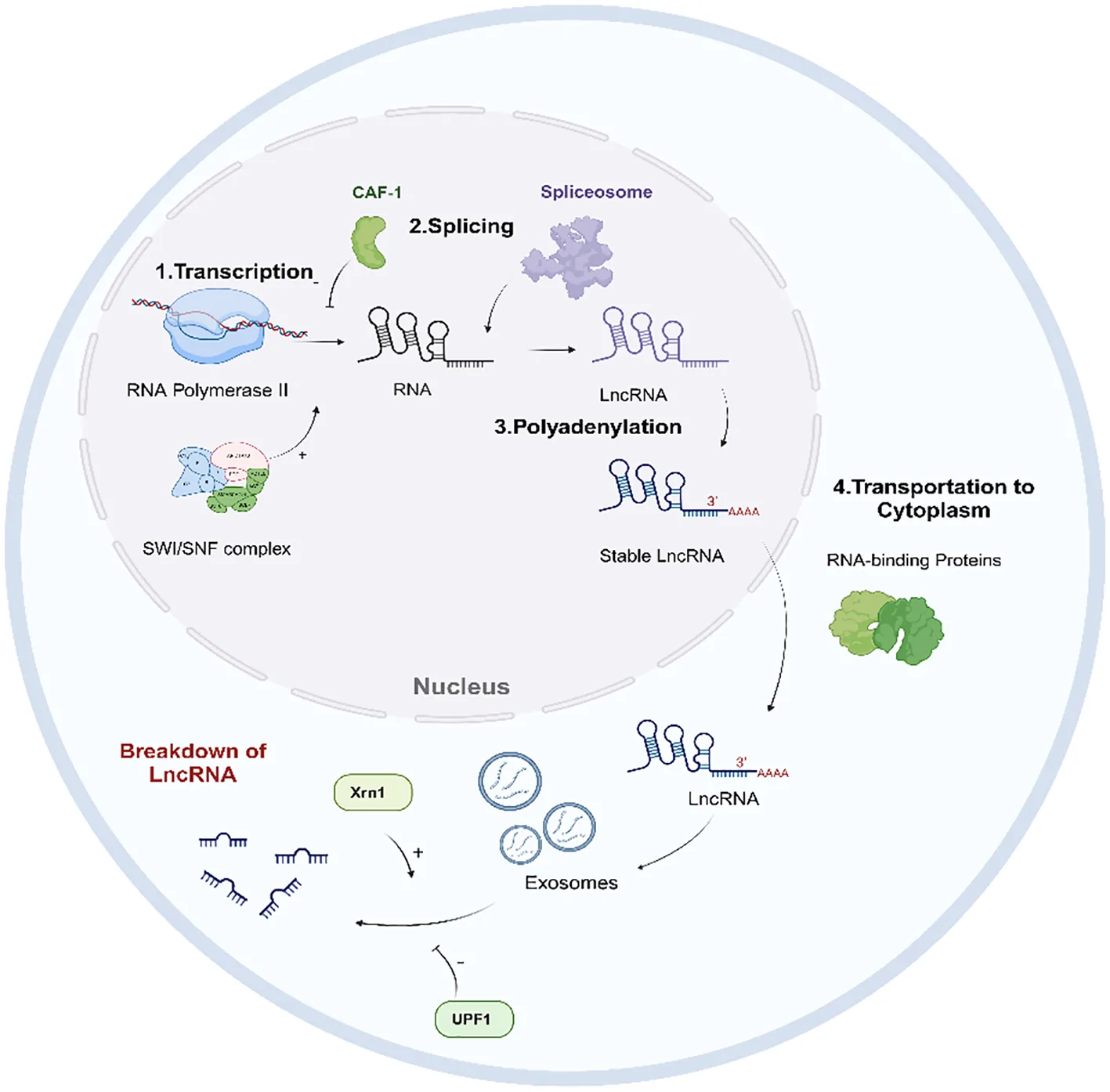
Biogenesis of lncRNAs. The process of lncRNA synthesis in the nucleus and its subsequent transport to the cytoplasm. 1) Transcription: The SWI/SNF chromatin remodeling complex and RNA Polymerase II start the transcription of lncRNA genes, producing a main RNA transcript. 2) Splicing: The spliceosome complex splices the original RNA transcript, eliminating introns to create a mature lncRNA molecule. 3) Polyadenylation: This process adds a poly (A) tail to the 3’ end of many mature lncRNAs, increasing their stability, facilitating nuclear export, and allowing them to operate correctly in the cell; however, polyadenylation is not universal, and some lncRNAs undergo alternative 3’ end processing mechanisms instead. 4) Transport to cytoplasm: The stable polyadenylated lncRNA is exported from the nucleus to the cytoplasm, where it may take part in regulatory activities, with the help of RNA-binding proteins. LncRNA Breakdown: Exosomes and exonucleases, such as Xrn1, can degrade lncRNAs. The stability of lncRNAs is also regulated by proteins such as UPF1, which can inhibit their breakdown, thus controlling their levels within the cell.

After transcription, precursor lncRNAs undergo 5’ capping for stability and translational recognition (55), followed by splicing, in which introns are excised, and exons joined (56). LncRNAs frequently undergo alternative splicing, producing multiple isoforms, and differ from mRNAs in having weaker splice signals, less efficient splicing, and more complex secondary structures; they also largely evade mRNA quality-control pathways like nonsense-mediated decay (57–59). Many mature lncRNAs are typically polyadenylated at the 3’ end, enhancing stability and enabling nuclear export (**Figure 2**) (59). However, polyadenylation is not a universal feature of lncRNAs. A substantial subset of lncRNAs, such as those processed by the RNase P/MRP-dependent pathway (e.g., MALAT-1) or stabilized by triple-helix-forming U1 snRNA-like structures (e.g., NEAT1), lack canonical poly(A) tails and instead rely on alternative 3’ end processing mechanisms for stability (59).

Export to the cytoplasm is mediated by RNA-binding proteins recognizing lncRNA-specific structures (55). Some lncRNAs act locally in the cytoplasm, while others remain nuclear to influence chromatin remodeling and gene regulation. For example, TUG1 recruits Polycomb Repressive Complex 2 (PRC2) in GBM, driving H3K27me3-mediated silencing of tumor suppressor genes and thereby promoting proliferation and invasion (60).

LncRNA biogenesis is further shaped by transcription factors and the epigenetic landscape (promoter accessibility, histone modifications, DNA methylation) (53, 55). For instance, H3K56 acetylation and the SWI/SNF complex promote antisense lncRNA transcription, while CAF-1 inhibits it (61). Degradation is mediated by nuclear exosomes (via the Nrd1-Nab3-Sen1 and TRAMP complexes) or cytoplasmic Xrn1, with UPF1 protecting lncRNAs from rapid turnover (54). Together, these findings highlight the central role of epigenetic regulation in lncRNA biogenesis (54).

Extrinsic stressors—including cellular stress, growth hormones, and environmental cues—can also modulate lncRNA biogenesis, enabling adaptive cellular responses (62). For example, MALAT-1 is upregulated under hypoxia, a common stressor in tumor microenvironments such as GBM, where it promotes angiogenesis, EMT-mediated migration, and survival pathways in stressed tumor cells (62). Understanding these biogenesis mechanisms not only clarifies the diverse regulatory roles of lncRNAs but also highlights their potential as therapeutic targets and biomarkers in GBM and other diseases (53, 63).

## 4 Mechanism of action of lncRNAs in GBM

**Figure 3:**
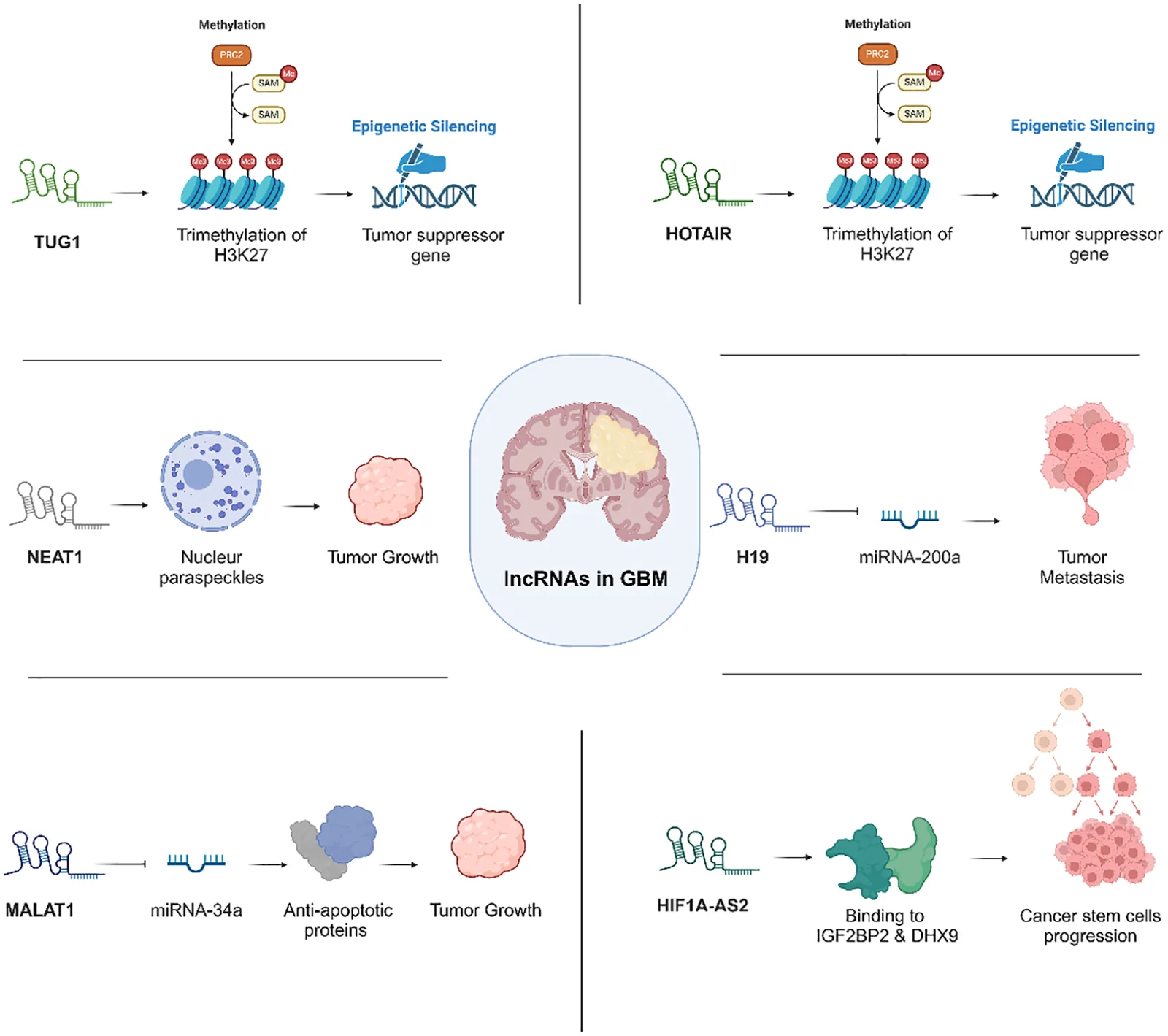
The role of lncRNAs in GBM. The molecular targets of many lncRNAs implicated in the development of GBM. Through PRC2-mediated trimethylation of H3K27, TUG1, and HOTAIR facilitate the silencing of tumor suppressor genes, resulting in epigenetic repression. NEAT1 may promote tumor development by forming nuclear paraspeckles. H19 and MALAT-1 interact with certain miRNAs, such as miRNA-200a and miRNA-34a, to promote tumor metastasis and proliferation, respectively. Finally, hypoxia-responsive genes are modulated by HIF1A-AS2, aiding cancer development.

LncRNAs have emerged as crucial regulators in the intricate gene expression networks that control cellular function and homeostasis (64). In GBM, the most aggressive and lethal form of brain cancer, lncRNAs play critical roles in driving oncogenic processes, such as uncontrolled cell proliferation, invasion, metastasis, and chemoresistance (**Table 2**, **Figure 3**) (64). These non-coding RNA molecules can regulate gene expression at multiple levels, including transcriptional, post-transcriptional, and epigenetic, through diverse mechanisms (64). One major pathway by which lncRNAs contribute to GBM pathogenesis is the hijacking of miRNAs, small non-coding RNAs that fine-tune gene expression by targeting specific mRNA transcripts for degradation or translational repression (63).

**Table 2:** The mechanism of action and function of different lncRNAs in GBM.

LncRNA	Function in GBM	Mechanism	References
MALAT1	Promotes GBM growth	Sponges miR-34a, increases anti-apoptotic proteins	(63)
HOTAIR	Attracts PRC2 to target genes, leading to epigenetic silencing, promotes oncogenesis	Interacts with chromatin-modifying enzymes	(65)
NEAT1	Essential for nuclear body formation, enhances tumor development and invasion	Influences RNA processing, stress response, and cell cycle progression	(66)
H19	Affects mRNA stability, translation, and localization which leads to enhanced tumor cell invasion and formation	Binds to miR-200a, stabilizes EMT-related mRNAs	(67)
TUG1	Enhances GBM advancement by suppressing genes that typically prevent cell proliferation and invasion	Attract PRC2 to certain gene loci, causing the deposition of H3K27me3 (a repressive histone mark)	(60)
HIF1A-AS2	Promotes cancer stem cell features in GBM	Binds with RNA-binding proteins (IGF2BP2 and DHX9) to increase target mRNA like HMGA1 stability.	(68)

Several studies have reported that miRNA expression is disrupted in GBM, with 256 miRNAs significantly overexpressed and 95 miRNAs downregulated, relative to normal brain tissue (69). Notably, miR-21, which is frequently regarded as carcinogenic, is upregulated in glioma cells and associated with higher tumor grades (70). Similarly, miR-221/222 are commonly elevated, whereas downregulation is associated with a favorable patient prognosis (70). MiR-181a/b/c are downregulated in GBM, with miR-181b/c acting as tumor suppressors and potential predictors of therapeutic response (71). MiR-182 was found to be overexpressed and linked to more aggressive tumors and worse survival rates (72). These uncontrolled miRNAs have important roles in glioma formation, prognosis, and treatment outcomes (69). Thus, they impede their interaction with target mRNAs, resulting in oncogene overexpression or tumor suppressor gene downregulation (69).

A critical function of lncRNAs in GBM is their ability to act as “molecular sponges” for miRNAs, a phenomenon known as competitive endogenous RNA (ceRNA) activity (63). In this mechanism, lncRNAs bind and sequester miRNAs, preventing them from interacting with their target mRNAs, thereby allowing uncontrolled expression of oncogenic genes (63). For example, the lncRNA MALAT-1 is known to promote GBM growth by sponging miR-34a, a tumor-suppressive miRNA (63). By inhibiting miR-34a, MALAT-1 promotes the accumulation of anti-apoptotic proteins, thereby enhancing cell survival and driving tumor growth (63).

Beyond miRNA sponging, lncRNAs can also exert regulatory effects on the epigenetic level (65). They often form ribonucleoprotein complexes with chromatin-modifying enzymes or transcription factors, thereby altering gene expression (65). For instance, the lncRNA HOTAIR has been extensively studied across various cancers, including GBM (65). HOTAIR acts by recruiting the PRC2, a group of proteins involved in epigenetic gene silencing, to specific genomic loci (65). This recruitment results in H3K27me3 deposition, which leads to the silencing of tumor-suppressor genes and promotes oncogenesis (65).

LncRNAs also act as molecular scaffolds, facilitating the formation of protein complexes that drive critical oncogenic signaling pathways (64). For example, NEAT1 (nuclear-enriched abundant transcript 1), which is involved in the formation of nuclear paraspeckles—nuclear bodies that regulate RNA processing, stress response, and other cellular processes (66). In GBM, NEAT1 has been shown to promote tumor progression by modulating extracellular matrix remodeling and advancing the cell cycle, thereby contributing to tumor growth and invasion (66). Another important mechanism by which lncRNAs influence cancer biology is through post-transcriptional gene regulation (60). LncRNAs can alter mRNA stability, translation efficiency, and localization, thereby fine-tuning protein production (60). For example, the lncRNA H19 has been shown to play a significant role in various cancers, including GBM, by modulating mRNA stability and translation (67). In GBM, H19 binds to miR-200a, a tumor-suppressive miRNA that targets mRNAs involved in EMT and invasion (67). By sponging miR-200a, H19 stabilizes these mRNAs, allowing for their continued expression, which promotes cell motility, invasion, and ultimately, tumor progression (67). This exemplifies how lncRNAs can contribute to the deregulation of key oncogenic pathways and promote the malignant phenotype in GBM (67).

Last but not least, lncRNAs are critical for cellular regulation, regulating gene expression and signaling pathways that regulate cell proliferation, differentiation, and survival (73). Certain lncRNAs in cancer can promote tumor growth by enhancing cancer stem cell characteristics, allowing cells to withstand treatment while maintaining their aggressive, stem-like traits (68). The mesenchymal stem-like characteristics of glioma stem cells (GSCs), which are associated with tumor aggressiveness, treatment resistance, and recurrence in GBM, are maintained in large part by the lncRNA HIF1A-AS2 (68). It has been demonstrated that IGF2BP2, a protein that binds directly to HIF1A-AS2, enhances the properties of GBM cancer stem cells by stabilizing key mRNAs such as HMGA1, particularly in hypoxic environments (68). HIF1A-AS2 elevates the activity of RNA-binding proteins, including DHX9 and IGF2BP2, thereby increasing the levels of their target proteins, including HMGA1 (68). Notably, HMGA1 is overexpressed in many cancers, and the severity of malignancy correlates with its levels (68). GBM patients’ elevated HMGA1 levels are linked to shorter progression-free survival, highlighting its role in aggressive tumor behavior (68).

Given the diverse and critical roles that lncRNAs play in GBM pathogenesis, they represent promising targets for novel therapeutic interventions (74). Efforts to therapeutically target lncRNAs in GBM could include antisense oligonucleotides (ASOs) to block their function, or small molecules designed to disrupt their interaction with miRNAs or protein complexes (74). Additionally, the development of delivery systems that can efficiently transport these therapies across the BBB and specifically target tumor cells is an area of active research (74).

## 5 Role of lncRNAs in GBM

Several lncRNAs are dysregulated in GBM, and their expression levels have been correlated with clinical parameters. LncRNAs play several roles in GBM, including cell proliferation, survival, invasion, cancer stem cell differentiation, and therapeutic resistance. Their effects are mediated through transcriptional, post-transcriptional, and epigenetic mechanisms, which remain only partially understood (75). Depending on their specific roles and regulatory mechanisms in GBM, these lncRNAs may function as oncogenes, promoting tumor development and progression, or as tumor suppressors, inhibiting cancer cell proliferation and tumor progression (76).

### 5.1 Oncogenic lncRNAs

Oncogenic lncRNAs play a critical role in GBM progression by modulating cellular proliferation and invasion (77). A dominant oncogenic mechanism in GBM is the ability of lncRNAs to function as miRNA sponges, thereby derepressing oncogenic targets and amplifying tumorigenic signaling. One way lncRNAs can indirectly influence gene expression is by functioning as miRNA sponges or competitive endogenous RNAs (ceRNAs) through binding to miRNAs (78). Several lncRNAs promote GBM progression through well-defined ceRNA axes. The lncRNA NEAT1 acts as a ceRNA, by binding to several miRNAs that suppress glioma cell growth and proliferation, including miR-139-5p, miR-132, miR-128-3p, miR-98-5p, and miR-107. This interaction consequently promotes GBM tumor cell proliferation and metastasis (79, 80). HOXA-AS2 enhances tumorigenesis by negatively regulating miR-885-5p and increasing RBBP4 expression (81). Similarly, HOXB-AS1 sponges miR-885-3p to regulate HOXB2 expression, thereby promoting proliferation and invasion. Furthermore, knockdown of HOXB-AS1 inhibits GBM cell proliferation by inducing S-phase cell cycle arrest and reducing their migratory and invasive capabilities (82). HOXC-AS3 regulates F11R (F11 receptor) by sequestering miR-216 and promotes malignant progression *in vivo* (83). RP3-439F8.1 promotes GBM via the miR-139-5p/NR5A2 (Nuclear Receptor Subfamily 5 Group A Member 2) axis (84), while Unigene56159 enhances proliferation and invasion by inhibiting miR-194-5p (85). Another GBM-overexpressed lncRNA is BCAR4, which is associated with higher tumor grades and poorer prognosis. Silencing BCAR4 inhibits cell growth and invasion, as well as tumor formation, by acting as a ceRNA to regulate miR-2276, thereby upregulating matrix metalloproteinase-7 (MMP7). This elucidates the critical role of the BCAR4/miR-2276/MMP7 signaling pathway in gliomagenesis, offering new insights into glioma treatment (86).

LPP-AS2 sponges miR-7-5p to regulate EGFR (epithelial growth factor receptor) and forms a positive feedback loop with c-MYC via the PI3K/AKT pathway (87). Inhibiting the lncRNA, LPP-AS2, reduces glioma cell proliferation and invasion while promoting apoptosis. LINC00152 suppresses miR-107, thereby upregulating HMGA2 (High-mobility group AT-hook 2) and promoting EMT (88). Its overexpression promotes cell proliferation and induces the expression of N-cadherin, Vimentin, and Snail, while downregulating E-cadherin, thereby facilitating EMT in GBM cells (88). On the other hand, LINC00645 regulates ZEB (Zinc finger E-box binding homeobox), an essential factor in EMT, via miR-205-3p under TGF-β influence (89). LINC01410, LINC01426, and MNX1-AS1 promote GBM tumorigenesis via miR-370-3p/PTEN (90), miR-345-3p/VAMP8 (vesicle-associated membrane protein 8) (91), and miR-4443 (92), respectively.

Another example is PVT1, which enhances cell proliferation and invasion while inhibiting apoptosis via miR-1301-3p/TMBIM6 (Transmembrane BAX Inhibitor Motif-containing 6) (93). While XIST promotes tumorigenesis by exhibiting multiple ceRNA roles through miR-448/ROCK1, miR-126/IRS1/PI3K/Akt, and reciprocal repression with miR-152 (94–96). It acts as a tumor promoter in GBM, and its knockdown triggers apoptosis, inhibits migration and proliferation, and diminishes glucose metabolism in tumor cells (95). LncRNA SOX2-OT is upregulated in GBM, and its silencing is associated with increased apoptosis. This lncRNA inhibits miRNA-192–5p, leading to overexpression of RAB2A, which activates the ERK pathway and subsequently enhances GBM cell proliferation (97). Furthermore, SOX2-OT contributes to TMZ insensitivity. It achieves this by recruiting ALKBH5, which mediates the demethylation of SOX2, leading to enhanced SOX2 expression, inhibition of apoptosis, increased cellular proliferation, and resistance to TMZ chemotherapy (98).

Additional ceRNA regulatory networks further illustrate how oncogenic lncRNAs promote GBM progression by sequestering miRNAs and derepressing critical oncogenic targets. LncRNA MIR4435-2HG enhances GBM growth and migration by downregulating miR-1224-5p, thereby upregulating TGFBR2 and contributing to tumor progression (99). Similarly, OXCT1-AS1 promotes GBM cell-cycle progression by inhibiting miR-195, thereby increasing CDC25A expression and enhancing the proliferative capacity of tumor cells (100).

SLC16A1-AS1 is upregulated in GBM and exerts oncogenic effects by suppressing miR-149 expression, thereby enhancing GBM cell growth and colony-forming ability and highlighting its diagnostic potential (101). HOXD-AS2 negatively regulates miR-3681-5p, which in turn modulates MALT1 expression. Knockdown of HOXD-AS2 reduces proliferation, migration, and invasion while promoting apoptosis *in vitro* and suppressing tumorigenesis in xenograft models (102).

RP1-86C11.7 contributes to GBM malignancy by sponging miR-144a-3p, preventing repression of TFRC (transferrin receptor) expression. This interaction increases intracellular free iron levels, thereby supporting tumor cell growth and survival (103). Likewise, RP11-390F4.3 promotes invasion and migration of GBM cells by simultaneously upregulating ROCK1 and suppressing miR-148a, establishing a regulatory axis that enhances tumor aggressiveness (104). The pro-angiogenic role of TUG1 in GBM is mediated by downregulation of miR-299, thereby increasing vascular endothelial growth factor A (VEGFA) expression and stimulating endothelial cell proliferation, migration, and tube formation (105). In the context of chemoresistance, OIP5-AS1 acts as a miR-129-5p sponge, upregulating IGF2BP2 (insulin-like growth factor 2 mRNA-binding protein 2) and thereby promoting proliferation while reducing sensitivity to temozolomide (TMZ). Silencing OIP5-AS1 or restoring miR-129-5p expression significantly enhances TMZ responsiveness (106). Furthermore, elevated lncRNA CRNDE expression has been observed in patients resistant to TMZ and correlates with their treatment responses. Silencing CRNDE enhances sensitivity to TMZ by reducing LC3 II/I, Beclin1, and Atg5 levels, while increasing p62 expression. This mechanism inhibits autophagy by activating the PI3K/Akt/mTOR pathway and is strongly correlated with ABCG2 expression (107). SOX2-OT contributes to TMZ resistance through ALKBH5-mediated SOX2 demethylation (98). HOTAIRM1 enhances radioresistance and modulates ROS and mitochondrial function (108). LINC01057 also increases radioresistance via NF-κB signaling (109).

TPT1-AS1 promotes GBM proliferation by sequestering miR-23a-5p and upregulating extracellular matrix protein 1 (ECM1), whereas PITPNA-AS1 enhances EGFR expression and activates the PI3K/AKT pathway through miR-223-3p inhibition (110) (111). FEZF1-AS1 exerts similar oncogenic effects by binding to miR-363-3p, thereby increasing the expression of the RNA-binding protein NOB1 and enhancing tumor progression (112). HOTAIRM1 participates in two distinct ceRNA axes: it regulates transglutaminase 2 (TGM2) via miR-17-5p and modulates ZEB2 through miR-873-5p, thereby influencing GBM progression, therapy resistance, and cellular survival mechanisms (108, 113). SNHG11 promotes EMT and invasive behavior by sponging miR-154-5p (114). VIM-AS1 regulates GBM proliferation and migration through the miR-105-5p/WEE1 (a tyrosine kinase that inhibits cyclin-dependent kinases) pathway, affecting key cell-cycle and survival proteins (115).

Finally, EWSAT1 enhances tumor growth and invasion by suppressing miR-152-3p (116), whereas FLVCR1-AS1 promotes proliferation and invasion by inhibiting miR-30b-3p, reinforcing its potential as both a diagnostic biomarker and a therapeutic target in GBM (117). Several oncogenic lncRNAs directly regulate cell proliferation and cell-cycle transitions in GBM. CASP5 overexpression enhances proliferation and invasion, while its knockdown induces G1 arrest and apoptosis (118). FER1L4 silencing reduces the viability and invasiveness of glioma cells while promoting apoptosis (119). HOXB-AS1 induces S-phase arrest upon knockdown (82). Silencing TALNEC2 leads to decreased cell proliferation, G1/S phase cell-cycle arrest, and reduced self-renewal and mesenchymal transformation in GSCs (120). Additionally, TALNEC2 knockdown alters the expression of specific miRNAs associated with cell cycle regulation and proliferation, notably miR-21 and miR-191, which mediate these effects. These findings underscore TALNEC2’s essential role in GBM progression (120). LncRNA DLEU1 expression is correlated with TRAF4 (TNF receptor-associated factor 4), which is likewise elevated in GBM tissues. Silencing DLEU1 reduces TRAF4 expression and diminishes GBM cell viability, indicating that both DLEU1 and TRAF4 contribute to GBM cell proliferation and may represent promising therapeutic targets (121).

Some lncRNAs exert oncogenic roles by modulating central GBM signaling cascades. MIR22HG is among the most dysregulated lncRNAs in GBM and correlates with poor patient prognosis. MIR22HG activates Wnt/β-catenin signaling by regulating SFRP2 (secreted frizzled related protein 2) and PCDH15 (protocadherin-15) via miR-22 derivatives (122). LPP-AS2 and PITPNA-AS1 activate the EGFR/PI3K/AKT/c-MYC axis (87, 111). LINC01410 downregulates PTEN, thereby activating Akt signaling (90). SNHG20 enhances stemness by activating the PI3K/Akt/mTOR pathway (123), while CRNDE regulates autophagy and TMZ sensitivity via the PI3K/Akt/mTOR pathway (107). LINC01057 acts as a tumor-promoting factor in GBM; its overexpression contributes to increased malignancy in tumor cells. LINC01057 promotes NF-κB activation and radioresistance by maintaining nuclear IKKα (109). SOX2-OT activates ERK signaling via RAB2A (97).

Multiple lncRNAs facilitate GBM invasiveness by regulating EMT. LINC00152 induces the expression of EMT markers, including N-cadherin, Vimentin, and Snail, while downregulating E-cadherin (88). SNHG11 promotes EMT via miR-154-5p (114). VIM-AS1 influences proliferation and migration by regulating WEE1 (115). HOXC-AS3 and BCAR4 also enhance invasion *in vivo* by regulating F11R and MMP7 (83, 86).

The overexpression of lncRNA LINC01503 in GBM was associated with the stemness of GBM. LINC01503 stabilizes GLI2 (GLI family zinc finger 2) by preventing its ubiquitination, thereby elevating stemness markers, including CD133, SOX2, NESTIN, ALDH1A1, and MSI1 (124). SNHG20 and RPSAP52 promote stem-like features through the PI3K/Akt/mTOR and TGF-β1 signaling pathways, respectively (123, 125). A cell stemness assay revealed that overexpression of RPSAP52 and TGF-β1 increased the number of CD133+ cells (125). TALNEC2 is essential for GSC self-renewal and mesenchymal transformation (120).

LncRNA PRADX is overexpressed in the mesenchymal subtype of GBM and is regulated by the RUNX1-CBFβ complex. Overexpressed PRADX inhibits BLCAP expression by interacting with EZH2 and catalyzing the trimethylation of lysine 27 on histone H3 (H3K27me3), which, in turn, influences STAT3 phosphorylation and promotes ACSL1 expression to accelerate cellular metabolism (126).

Collectively, these oncogenic lncRNAs illustrate that a predominant mechanism in GBM is ceRNA-mediated miRNA sequestration, which relieves repression of key oncogenic targets that control proliferation, invasion, angiogenesis, metabolism, stemness, and therapy resistance. Through these axes, lncRNAs converge on central pathways, including EGFR/PI3K/AKT, Wnt/β-catenin, NF-κB, ERK, and regulators of EMT, the cell cycle, and iron homeostasis, thereby amplifying malignant phenotypes. A consolidated summary of these lncRNA/miRNA/mRNA regulatory networks and their molecular pathways in GBM is provided in **Table 3**.

**Table 3:** Oncogenic lncRNAs and their target pathways in GBM.

LncRNA name	Molecular pathway	References
BCAR4	Functions as a ceRNA that regulates miR-2276, subsequently leading to the upregulation of MMP7	(86)
CRNDE	CRNDE Silencing reduces the levels of LC3 II/I, Beclin1, and Atg5, while increasing p62 expression, and activate the PI3K/Akt/mTOR pathway	(107)
DLEU1	Positively regulatesTRAF4 expression	(121)
EWSAT1	Decreases the expression level of miR-152–3p	(116)
FEZF1-AS1	Binds to miR-363-3p, leading to the upregulation of NOB1 as a downstream target	(112)
FLVCR1-AS1	Serves as an oncogene by inhibiting miR-30b-3p	(117)
HOTAIRM1	Sponges hsa-mir-17-5p and thereby increases TGM2 transcript and protein levels. Part of the regulatory axis HOTAIRM1/miR-873-5p/ZEB2	(108), (113)
HOXA-AS2	HOXA-AS2 negatively regulates miR-885-5p, leading to an increase in RBBP4 expression	(81)
HOXB-AS1	Functions as a ceRNA by sponging miR-885-3p, influences the expression of HOXB2	(82)
HOXC-AS3	Act as ceRNA that sponges miR-216, and regulates the expression of the F11R	(83)
HOXD-AS2	Negatively regulate mir-3681-5p, and promoting the expression of MALT1	(102)
LINC00152	Suppresses mir-107 expression in U87 cells, leading to an increase in HMGA2	(88)
LINC00645	with miR-205-3p, regulating the expression of ZEB1	(89)
LINC01057	Maintains the nuclear localization of ikkα, thereby facilitating NF-κb activation, which ultimately drives GBM progression	(109)
LINC01410	Inhibit miRNA-370-3p, subsequently leading to the downregulation of PTEN and facilitating tumorigenesis via Akt signaling pathways	(90)
LINC01426	Sequesters miR-345-3p, which in turn elevates the levels of VAMP8, an oncogenic coding gene that drives GBM progression	(91)
LINC01503	Enhances the stability of GLI2 by inhibiting its ubiquitination by FBXW1, thereby elevating the levels of CD133, SOX2, NESTIN, ALDH1A1, and MSI1, which collectively contribute to the stem-like properties of GBM	(124)
LPP-AS2	Functions as a ceRNA that regulates EGFR expression by sponging miR-7-5p	(87)
MIR22HG	Targets miR-22-3p and miR-22-5p, thereby modulating their direct targets and subsequently influencing the Wnt/β-catenin signaling pathway.	(122)
MIR4435-2HG	Downregulates the expression of miRNA-1224-5p, which in turn leads to the upregulation of TGFBR2 and contributes to the progression of GBM	(99)
MNX1-AS1	Enhances GBM cell proliferation, migration, and invasion by suppressing miR-4443	(92)
OIP5-AS1	OIP5-AS1 acts as a sponge for miR-129-5p, which in turn targets IGF2BP2	(106)
OXCT1-AS1	Regulate the miR-195-5p/CDC25A axis	(100)
PITPNA-AS1	Sequesters microRNA-223-3p, thereby regulating the expression of EGFR and activating the PI3K/AKT signaling pathway	(111)
PRADX	Inhibits BLCAP expression by interacting with EZH2 and catalyzing H3K27me3, which, in turn, influences STAT3 phosphorylation and promotes ACSL1 expression	(126)
PVT1	Elevates TMBIM6 expression through miR-1301-3p.	(93)
RP1-86C11.7	Regulates TFRC levels by sponging the microRNA hsa-miR-144a-3p	(103)
RP11-390F4.3	Positively regulates ROCK1 and negatively regulates miR-148a.	(104)
RP3-439F8.1	Acts as a ceRNA that NR5A2 by sponging the microRNA miR-139-5p	(84)
RPSAP52	Positively regulates TGF-β1 expression	(125)
SLC16A1-AS1	Reduces the expression of miRNA-149, thereby enhancing the growth rate and colony-forming capacity of GBM cells	(101)
SNHG11	Negatively regulates of miR-154-5	(114)
SNHG20	Activates the PI3K/Akt/mtor signaling pathway	(123)
SOX2-OT	Inhibits miRNA-192–5p, leading to the overexpression of RAB2A, which activates the ERK pathway. SOX2-OT resist TMZ by recruiting ALKBH5, which mediates the demethylation of SOX2, leading to enhanced SOX2 expression	(97) (98)
TALNEC2	Modifies the expression of specific miRNAs associated with cell cycle regulation and proliferation, notably involving miR-21 and miR-191 in mediating its effects.	(120)
TPT1-AS1	Act as a sponge for miR-23a-5p in GBM, promoting proliferation by upregulating ECM1	(110)
TUG1	Downregulate miR-299, which in turn modulate VEGFA levels	(105)
Unigene56159	Sequesters miR-194-5p	(85)
VIM-AS1	Downregulates miR-105-5p, and VIM-AS1 and miR-105-5p collaboratively regulate WEE1 expression	(115)
XIST	Inhibit miRNA-448, which in turn upregulates ROCK1, thereby enhancing cellular malignancy. Inhibits miRNA-126, thereby activating the IRS1/PI3K/Akt signaling pathway	(94) (95)

### 5.2 Tumor suppressor lncRNAs

Research has shown that overexpression of growth arrest-specific 5 (GAS5) inhibits tumor growth in glioma cell lines, including U87 and U251 (127). In GBM, miR-196a-5p is overexpressed (128), and GAS5 directly targets this oncomiR, thereby increasing FOXO1 levels, which in turn upregulate the tumor-suppressor genes PID1 (phosphotyrosine interaction domain-containing 1) and MIIP (migration and invasion inhibitory protein). FOXO1 also enhances GAS5 transcription, creating a positive feedback loop. Through the GAS5/miR-196a-5p/FOXO1 axis, GAS5 effectively restricts GSC proliferation, migration, and invasion while promoting apoptosis, highlighting its potential as a therapeutic target in glioma (129).

MEG3 is a potential biomarker and prognostic indicator in glioma. MEG3 is often downregulated in glioma tissues and cells, and its overexpression suppresses cell proliferation, migration, and invasion. miR-96-5p is a promising target of MEG3; the MEG3/miR-96-5p/MTSS1 (metastasis suppressor 1) signaling pathway could be a putative therapeutic target for glioma treatment. Also, overexpression of MEG3 abolished the effects of miR-93/MTSS1 signaling in promoting glioma cell growth and metastasis (130). MEG3 also acts as a ceRNA for miR-19a to inhibit tumorigenesis (131) and reduces MDM2 (Mouse double minute 2), thereby enhancing p53 stability. It acts through both p53-dependent and independent pathways to limit tumor growth (132).

The lncRNA CASC2, located on chromosome 10q26, is frequently downregulated in glioma tissues and has shown potential to improve the efficacy of TMZ, a standard chemotherapy for glioma. Exogenous CASC2 inhibits glioma cell proliferation and amplifies the repression of cell growth induced by TMZ. CASC2 overexpression increases TMZ sensitivity in resistant glioma cells by directly targeting miR-181a, which leads to PTEN upregulation and reduced p-AKT levels. These findings suggest CASC2 as a promising target for improving glioma treatment and as a potential diagnostic biomarker (133).

Mediator of DNA damage checkpoint protein 1 antisense RNA (MDC1-AS) is the antisense transcript of the tumor suppressor gene MDC1. Research in glioma cell lines (U87MG, U251, and HEB) and glioma tissues reveals significant downregulation of both MDC1-AS and MDC1. Overexpression of MDC1-AS notably inhibits cell-cycle progression and proliferation, indicating its potential tumor-suppressive role. A positive correlation between MDC1-AS and MDC1 expression was observed, as MDC1-AS acts as a tumor suppressor by upregulating the antisense tumor-suppressing gene MDC1 (134).

A study highlights BCYRN1 as a lncRNA that is significantly downregulated in glioma and correlates its low expression with poor patient outcomes. Overexpression of BCYRN1 suppresses glioma cell growth and migration, whereas BCYRN1 depletion has the opposite effect. Mechanistically, BCYRN1 sequesters miR-619-5p, thereby regulating CUEDC2 and the PTEN/AKT/p21 pathway to inhibit tumor progression. These findings suggest BCYRN1 as a promising diagnostic biomarker and therapeutic target for glioma management (135).

Tumor suppressor in lung cancer-1 antisense-1 (TSLC1-AS1) acts as the antisense transcript of the tumor suppressor gene TSLC1. In glioma, TSLC1-AS1 overexpression increases TSLC1 levels and significantly reduces U87 cell proliferation, migration, and invasion, while its knockdown in SNB-19 cells has the opposite effect. TSLC1-AS1 expression positively correlates with tumor suppressors (NF1, VHL, PIK3R1) and negatively with the oncogene BRAF, suggesting that TSLC1-AS1 functions as a tumor suppressor and a key mediator of TSLC1 expression in glioma (136). Tumor suppressor candidate 7 (TUSC7) is an antisense RNA gene that is notably downregulated in GBM and has been shown to suppress glioma malignancy. Research reveals that low TUSC7 expression correlates with increased TMZ resistance in GBM cells. Overexpressing TUSC7 in TMZ-resistant U87TR cells reduces the IC50 of TMZ, decreases multidrug resistance protein 1 (MDR1) levels (which lead to increased drug efflux and reduced intracellular drug concentration), and enhances TMZ cytotoxicity by directly targeting and silencing miR-10a. TUSC7 expression may serve as a prognostic marker and therapeutic target in glioma (137).

**Figure 4:**
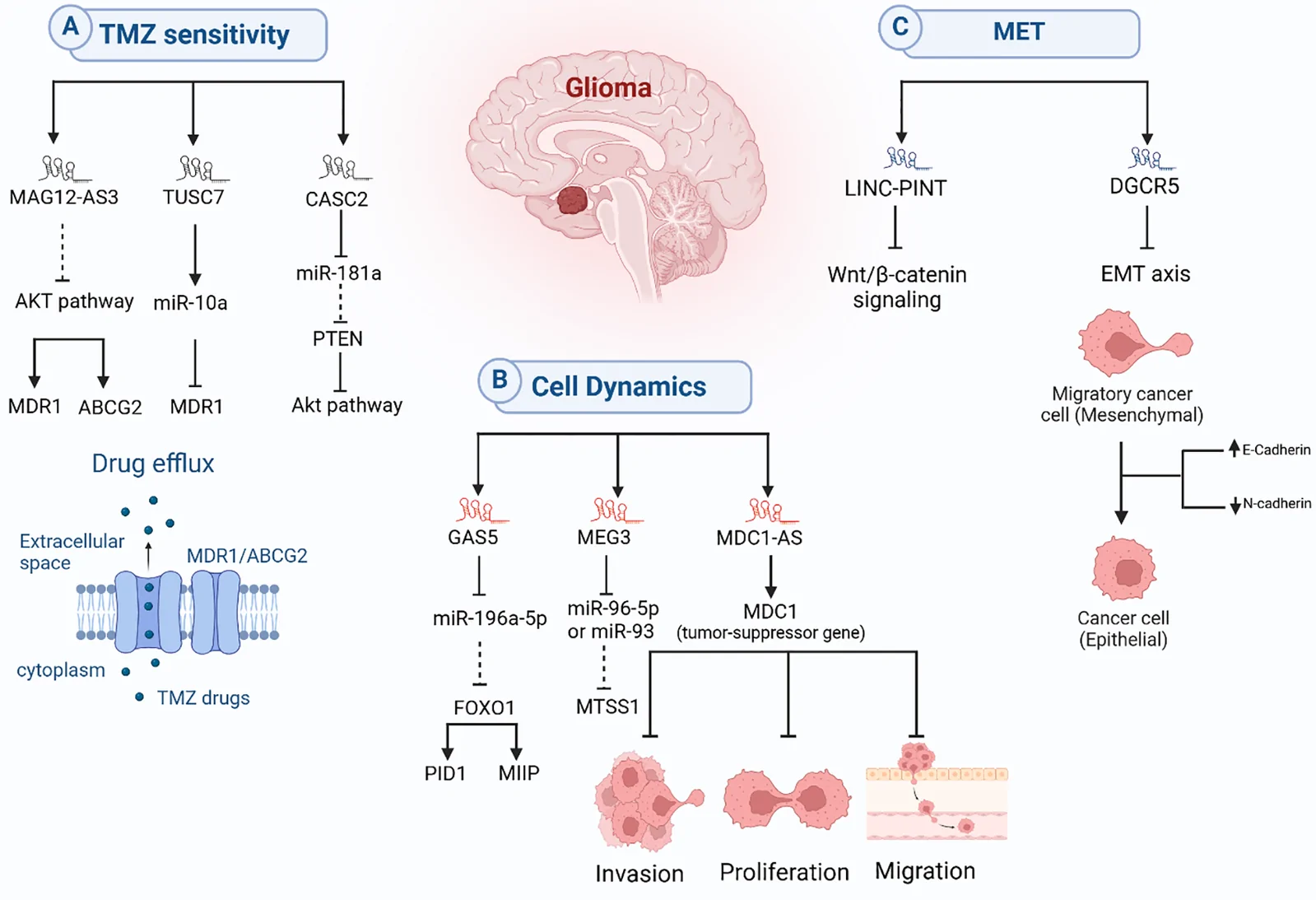
Prominent examples of tumor suppressor lncRNAs and their mechanisms of action in GBM. Several tumor-suppressor lncRNAs in GBM exert their effects through distinct mechanisms that influence drug sensitivity, cell dynamics, and cell-state transitions. **(A)** TMZ Sensitivity: lncRNAs such as MAGI2-AS3 and TUSC7 enhance sensitivity to TMZ by targeting drug efflux pathways. MAGI2-AS3 downregulates the AKT pathway, leading to decreased expression of the MDR1 and ABCG2 efflux pumps, thereby facilitating drug retention in the cell. Similarly, TUSC7 targets miR-10a to reduce MDR1 expression, while CASC2 regulates miR-181a/PTEN to inhibit the Akt pathway, promoting TMZ sensitivity. The Drug Efflux panel illustrates MDR1 and ABCG2 efflux pumps at the cell membrane, which export TMZ drugs, contributing to drug resistance. Suppression of these pumps by tumor-suppressor lncRNAs enhances drug retention, thereby supporting drug efficacy. **(B)** Cell Dynamics: Tumor-suppressor lncRNAs, including GAS5, MEG3, and MDC1-AS, regulate various cellular behaviors implicated in cancer progression. GAS5 inhibits miR-196a-5p, leading to the activation of FOXO1, which upregulates tumor-suppressive genes PID1 and MIIP to suppress invasion. MEG3 targets miR-96-5p or miR-93, thereby activating MTSS1 and affecting cell proliferation and migration to limit GBM progression. MDC1-AS modulates MDC1 (a tumor suppressor gene), thereby influencing cell proliferation and migration and reducing GBM aggressiveness. **(C)** MET: lncRNAs LINC-PINT and DGCR5 are shown to influence cancer cell state transitions. LINC-PINT suppresses Wnt/β-catenin signaling to promote epithelial characteristics, while DGCR5 modulates the EMT axis, affecting markers such as E-cadherin and N-cadherin to regulate mesenchymal and epithelial cell states. GBM, glioblastoma; lncRNA, long non-coding RNA; TMZ, temozolomide; PTEN, phosphatase and tensin homolog; MDR1, multidrug resistance protein 1; ABCG2, ATP-binding cassette sub-family G member 2; MDC1, Mediator of DNA Damage Checkpoint 1; FOXO1, forkhead box O1; PID1, phosphotyrosine interaction domain containing 1; MIIP, migration and invasion inhibitory protein; MTSS1, metastasis suppressor 1; EMT, epithelial-mesenchymal transition; MET, mesenchymal-to-epithelial transition; AKT, protein kinase B.

ADAM metallopeptidase with thrombospondin type 1 motif, 9 antisense RNA 2 (ADAMTS9-AS2) is a lncRNA and the antisense transcript of the tumor suppressor gene ADAMTS9 (138). Research indicates that low ADAMTS9-AS2 expression is an independent predictor of poor survival in glioma. Overexpression of ADAMTS9-AS2 significantly inhibits glioma cell migration, while its knockdown promotes migration. Additionally, ADAMTS9-AS2 expression is negatively correlated with DNA methyltransferase-1 (DNMT1), as DNMT1 knockdown and treatment with 5-aza-dC increase ADAMTS9-AS2 expression. Thus, ADAMTS9-AS2 may serve as a DNMT1-regulated tumor suppressor, as well as a potential biomarker and therapeutic target, in glioma (138). **Figure 4** and **Table 4** summarize additional lncRNAs implicated in GBM, providing an overview of their functions and relevance in this aggressive malignancy. This compilation aims to enhance our understanding of the lncRNA landscape in GBM and underscores the need for further research into their roles as potential biomarkers and therapeutic agents.

**Table 4:** Tumor suppressor lncRNAs and their target pathways in GBM.

LncRNA	Expression pattern	Target	Role in GBM	References
GAS5	Downregulated	miR-196a-5p/FOXO1 axis.	Inhibits proliferation, migration, and invasion of GBM	(129)
MEG3	Downregulated	-miR-96-5p or miR-93/MTSS1 Axis - miR-19a/MDM2/p53 Axis	Inhibits glioma cell proliferation	(130, 131)
CASC2	Downregulated	miR-181a/PTEN/Akt axis.	Inhibits proliferation and increases TMZ sensitivity	(133)
MDC1-AS	Downregulated	upregulates antisense tumor-suppressing gene MDC1	Tumor suppressor; suppresses the cell cycle and proliferation.	(134)
BCYRN1	Downregulated	miR-619-5p/CUEDC2 and the PTEN/AKT/p21 axis.	Inhibits glioma tumorigenesis and progression	(135)
TSLC1-AS1	Downregulated	Upregulates the tumor suppressor gene TSLC1	Inhibits cell proliferation, migration, and invasion.	(136)
TUSC7	Downregulated	miR-10a/MDR1 axis.	Inhibits TMZ resistance and tumor malignancy and helps prevent drug efflux	(137)
ADAMTS9-AS2	Downregulated	Regulates DNMT1 (DNA methyltransferase 1)	Suppresses cell migration	(138)
CASC7	Downregulated	Wnt/β-catenin signaling pathway	Inhibits the progression of glioma.	(139)
PART1	Downregulated	PART1/miR-190a 3p/PTEN/PI3K/AKT axis	Suppresses glioma progression	(140)
AC016405.3	Downregulated	miR-19a-5p/TET2 axis	Reduces GBM cell proliferation and invasion	(141)
MATN1-AS1	Downregulated	RELA/MAPK axis	Suppresses GBM cell from proliferation and invasion, and promoting apoptosis through the downregulation of survival proteins	(142)
LINC00657	Downregulated	LINC00657/miR-190a-3p axis	Reduces tumor growth and progression in GBM	(143)
RNCR3	Downregulated	miR-185-5p/KLF16 axis (Krüppel-like factor 16)	Reduces tumor growth and progression in GBM	(144)
NBAT1	Downregulated	NBAT1/Akt axis	Reduces the malignancy and prognosis of gliomas	(145)
RAMP2-AS1	Downregulated	NOTCH3/DHC10 axis	Inhibits GBM cell growth while supporting normal cell cycle progression	(146)
RP11-838N2.4	Downregulated	RP11-838N2.4/miR-10a/EphA8 axis, along with inhibiting TGF-β signaling	Lower levels are associated with a higher risk of GBM relapse and shorter survival times.	(147)
LINC-PINT	Downregulated	Wnt/β-catenin signaling.	Suppresses cell growth, invasion, and EMT in GBM	(148)
PWRN1	Downregulated	miR-21-5p/PDCD4 axis	Suppresses GBM cancer cell growth and migration	(149)
UNC5B-AS1	Downregulated	miR-24-3p/Maxi 1 axis	Suppresses cell proliferation.	(150)
MAGI2-AS3	Downregulated	Akt/MDR1 and ABCG2 Axis	Enhances apoptosis, tumor Suppression in Glioma Cells and reverse TMZ resistance through preventing drug efflux	(151)
DGCR5	Downregulated	EMT Axis	Increases epithelial markers (e.g., E-cadherin) and decreases mesenchymal markers (e.g., N-cadherin), reversing EMT and reducing invasiveness	(152)
LINC00294	Downregulated	miR-1278/NEFM Axis	Suppresses glioma cell proliferation.	(153)
AF127577.4	Downregulated	AF127577.4-ORF/ERK2/METTL3 Axis	The micropeptide AF127577.4-ORF, encoded within a lncRNA, reduces GBM cell proliferation.	(154)

GAS5, Growth arrest-specific 5; MEG3, Maternally expressed gene 3; CASC2, Cancer susceptibility candidate 2; MDC1-AS, mediator of DNA damage checkpoint protein 1antisense RNA; BCYRN1, Brain Cytoplasmic RNA 1; TSLC1-AS1, Tumor suppressor in lung cancer-1 antisense-1; TUSC7, Tumor suppressor candidate 7; ADAMTS9-AS2, ADAM metallopeptidase with thrombospondin type 1 motif, 9 antisense RNA 2; CASC7, cancer susceptibility candidate 7; PART1, prostate androgen-regulated transcript 1; MATN1-AS1, Matrilin-1 Antisense RNA 1; LINC00657, Long Intergenic Non-Protein Coding RNA 657; RNCR3, retinal noncoding RNA3; NBAT1, neuroblastoma associated transcript 1; RAMP2-AS1, Receptor Activity Modifying Protein 2-Antisense RNA 1; LINC-PINT, Long Intergenic Non-Protein Coding RNA, p53 Induced Transcript; PWRN1, Prader-Willi region non-protein coding RNA 1; UNC5B-AS1,unc-5 netrin receptor B antisense RNA 1; MAGI2-AS3, membrane-associated guanylate kinase inverted-2 antisense RNA 3; DGCR5, DiGeorge syndrome critical region gene 5; FOXO1, forkhead box O1; PID1, phosphotyrosine interaction domain containing; MIIP, migration and invasion inhibitory protein; MTSS1, metastasis suppressor 1; EMT, epithelial-mesenchymal transition; MET, mesenchymal-to-epithelial transition; AKT, protein kinase B.

Although numerous lncRNAs have been identified in GBM and their molecular functions have been extensively summarized in previous reviews, recent evidence suggests that the field has moved beyond characterizing individual lncRNAs and their downstream targets (24, 155). Rather than acting through isolated molecular pathways, many lncRNAs converge on common biological programs that regulate glioma stem cell maintenance, metabolic reprogramming, epithelial–mesenchymal transition, immune evasion, and therapeutic resistance. This suggests that lncRNAs function as components of highly interconnected regulatory networks rather than independent oncogenic drivers (53).

Furthermore, advances in single-cell RNA sequencing and spatial transcriptomics have begun to reveal the context-dependent expression of lncRNAs across distinct glioblastoma cell states and tumor regions, highlighting their potential contribution to intratumoral heterogeneity and disease progression (156). Emerging evidence further suggests that lncRNAs contribute to communication between glioblastoma cells and the tumor microenvironment, including interactions with tumor-associated macrophages, microglia, endothelial cells, and other stromal components, which collectively influence tumor progression, immunosuppression, angiogenesis, and therapeutic response (157).

Despite these advances, the translation of lncRNA discoveries into clinical practice remains limited by challenges including intratumoral heterogeneity, insufficient clinical validation, inefficient delivery across the blood-brain barrier, and the lack of standardized therapeutic strategies (158). By integrating these emerging concepts with established molecular mechanisms, this review provides a critical synthesis of current knowledge and highlights the opportunities and remaining challenges for exploiting lncRNAs as biomarkers and therapeutic targets in precision medicine for GBM.

### 5.3 LncRNAs in glioma stem cells

Glioma stem cells (GSCs) constitute a highly tumorigenic subpopulation within glioblastoma that possesses self-renewal capacity, multilineage differentiation potential, and the ability to initiate tumor formation. Increasing evidence indicates that GSCs are major drivers of GBM initiation, intratumoral heterogeneity, diffuse tissue infiltration, therapeutic resistance, and tumor recurrence following surgery and chemoradiotherapy (159–162). Compared with differentiated glioma cells, GSCs exhibit enhanced DNA damage repair, metabolic plasticity, resistance to apoptosis, and preferential localization within specialized hypoxic and perivascular niches that preserve their stem-like phenotype and promote resistance to TMZ and radiotherapy (159, 160). Their remarkable capacity to survive conventional therapies and regenerate recurrent tumors has made GSC eradication a major objective in the development of effective GBM treatments. Recent transcriptomic, functional, and single-cell RNA sequencing (scRNA-seq) studies have revealed that these defining characteristics are orchestrated by a specialized repertoire of GSC-enriched lncRNAs, which regulate stemness-associated transcription factors, epigenetic programs, post-transcriptional networks, and metabolic adaptation (25, 163, 164). These lncRNAs collectively govern GSC self-renewal, phenotypic plasticity, adaptation to microenvironmental stress, and resistance to therapy.

One of the best-characterized GSC-associated lncRNAs is HIF1A-AS2, which is selectively enriched in mesenchymal GSCs residing within hypoxic niches. HIF1A-AS2 functions as a molecular scaffold that interacts with the RNA-binding proteins IGF2BP2 (Insulin-like growth factor 2 mRNA-binding protein 2) and DHX9 (DExH-box Helicase 9), enhancing the stability of oncogenic transcripts such as HMGA1 and promoting adaptation to hypoxia, maintenance of stemness, and tumor progression (68). Similarly, LINC01503 promotes GSC stemness by preventing ubiquitin-mediated degradation of GLI2 (GLI Family Zinc Finger 2), thereby increasing the expression of canonical stem cell markers, including CD133, SOX2, NESTIN, ALDH1A1, and MSI1 (124). Another important regulator, TALNEC2, suppresses differentiation while promoting GSC self-renewal, mesenchymal transformation, and radioresistance. TALNEC2 silencing inhibits stem cell maintenance, enhances radiosensitivity, and prolongs survival in orthotopic xenograft models, partly through regulation of miR-21 and miR-191 (120). Additional oncogenic lncRNAs, including SNHG20 and RPSAP52, further sustain GSC maintenance by activating the PI3K/AKT/mTOR and TGF-β signaling pathways, respectively (123).

Beyond regulating stemness, several lncRNAs contribute directly to therapeutic resistance. The paraspeckle-associated lncRNA NEAT1 is markedly upregulated in CD133+/CD44+ GSCs and functions as a ceRNA that sequesters let-7g-5p, thereby derepressing MAP3K1 signaling and conferring resistance to TMZ-induced apoptosis (165). More broadly, GSC-enriched lncRNAs coordinate cellular responses to chemotherapy, radiotherapy, hypoxia, and metabolic stress by regulating apoptosis, DNA repair pathways, and adaptive signaling networks, thereby enabling treatment-resistant GSC populations to survive and drive tumor recurrence.

Conversely, several tumor-suppressive lncRNAs antagonize GSC maintenance and tumorigenicity. GAS5 suppresses GSC proliferation, migration, and invasion through the miR-196a-5p/FOXO1 signaling axis, thereby limiting stemness and reducing tumor aggressiveness (129). Likewise, restoration of MEG3 expression inhibits glioma cell proliferation and stem-like characteristics by regulating multiple tumor suppressor pathways, including the miR-96-5p/MTSS1 and p53 signaling axes (130). These findings highlight that lncRNAs can function either as oncogenic drivers or tumor suppressors depending on their molecular context within the GSC regulatory network (166).

Given their central role in regulating self-renewal, therapeutic resistance, and tumor recurrence, GSC-enriched lncRNAs represent attractive therapeutic targets. Emerging RNA-based strategies, including antisense oligonucleotides, RNA interference, CRISPR-mediated gene modulation, and nanoparticle-based delivery systems that can cross the blood-brain barrier, offer promising approaches for selectively targeting oncogenic lncRNAs while preserving normal neural stem cells. Furthermore, advances in single-cell RNA sequencing and spatial transcriptomics are expected to facilitate the identification of GSC-specific lncRNA signatures, enabling precision therapies directed toward eliminating treatment-resistant stem cell populations (25).

## 6 The role of lncRNA as diagnostic and prognostic biomarkers in GBM

LncRNAs have gained attention as valuable molecular markers for diagnosing GBM and predicting patient outcomes. With distinct expression patterns in cancerous and normal tissues and their stability in biofluids, lncRNAs offer a minimally invasive approach for molecular diagnostics and prognostic assessments, potentially enhancing clinical outcomes by supporting early diagnosis before clinical manifestation.

### 6.1 Diagnostic lncRNAs in GBM

Serum HOTAIR levels were significantly higher in GBM patients than in normal controls (167). This finding indicates the potential of HOTAIR as a serum diagnostic biomarker for GBM. Further supporting this potential is the finding by Shen et al. that high HOTAIR inlevels are associated with a 2.04-fold decrease in mortality (168). Interestingly, higher NEAT1 levels were strongly associated with larger tumor sizes, advanced WHO grades, and recurrence in GBM tissues (169). Further, Wu et al. observed elevated NEAT1 expression levels in the plasma of GBM patients (170). Additionally, patients receiving postoperative chemoradiotherapy with high NEAT1 expression had poorer prognoses, indicating a role for NEAT1 in the proliferation and metastasis of high-grade gliomas and suggesting the potential for new NEAT1-targeted treatments (169).

GAS5 serves as a serum diagnostic biomarker because it is downregulated in numerous tumors, including ovarian and breast cancers, non-small cell lung cancers, large B-cell lymphoma, and gastric and laryngeal cancers (171, 172). However, in glioma, GAS5 inhibits cell proliferation and growth by acting as a ceRNA that targets several miRNAs, including miR-222, miR-10b, and miR-196a-5p (127, 129). Specifically, Toraih et al. observed a significant reduction in lncRNA GAS5 expression in GBM tissues (173). In addition, Shen et al. reported that elevated serum GAS5 levels were associated with a 56% reduction in mortality risk among GBM patients. This suggests the potential application of lncRNA GAS5 as a diagnostic and prognostic biomarker in GBM (168).

The SAMMSON (Survival Associated Mitochondrial Melanoma-Specific Oncogenic Non-Coding RNA) gene is located on chromosome 3p13–3p14 and is recognized for its oncogenic role in various cancers, including melanoma, liver cancer, gastric cancer, papillary thyroid carcinoma, and oral squamous cell carcinoma (174, 175). However, in GBM cells, Ni et al. observed elevated expression of the lncRNA SAMMSON (176). Additionally, SAMMSON functions as a ceRNA by binding to miR-622, thereby promoting GBM cell proliferation (176). Furthermore, Xie et al. reported that lncRNA SAMMSON was significantly overexpressed in the plasma of GBM patients compared to normal controls, supporting its utility as a diagnostic biomarker (176).

### 6.2 Prognostic lncRNAs in GBM

LINC00565 and LINC00641 were identified to be highly elevated in liquid biopsies of GBM patients, showing strong correlations with tumor size and patient survival. These biomarkers exhibited high diagnostic sensitivity and specificity, indicating their potential as non-invasive prognostic markers, particularly as elevated expression levels are associated with poorer progression-free survival (PFS) and overall survival. Furthermore, both LINC00565 and LINC00641 have been implicated in pathways that drive tumor growth and metastasis, with higher levels of these lncRNAs linked to more aggressive disease. These findings highlight the potential utility of LINC00565 and LINC00641 as liquid biopsy markers for GBM prognosis and diagnosis (177).

Also, the lncRNA TP73-AS1 has been linked to GBM aggressiveness and has been shown to promote stemness in GBM cells and mediate TMZ resistance in GBM cancer stem cells. Thus, this lncRNA serves as an effective prognostic marker of GBM progression and chemotherapy resistance (176). This finding is supported by a study by Dai et al., which showed that patients with high TP73-AS1 levels exhibited increased resistance to TMZ, indicating that TP73-AS1 is a valuable prognostic indicator (177).

Compared with other invasive GBM diagnostic methods, circulating genetic biomarkers are easier, faster, and more cost-effective. These biomarkers offer a promising approach to diagnosis, treatment, and prognosis, and to avoiding unnecessary chemotherapy by early identification of potential chemoresistance. LncRNAs have shown a strong correlation with GBM diagnosis, prognosis, and tumor grading, suggesting additional mechanisms underlying chemoresistance that require further investigation. This opens up the potential for these biomarkers to be used clinically to reduce the need for invasive procedures, aid in diagnosing and treating patients with chemoresistant GBM, and target specific lncRNAs associated with chemoresistance. LncRNAs have proven to be valuable for diagnostic and prognostic applications. Excessive experimental and clinical studies are necessary to identify the most sensitive and specific markers associated with high plasma levels in GBM subtypes and aggressiveness, and to explore the feasibility of using lncRNAs as a quick, easy, and noninvasive diagnostic and therapeutic approach for GBM. Incorporating lncRNAs into diagnostic and prognostic strategies may improve GBM patient care by enhancing precision medicine. The use of lncRNA-based biomarkers for both early detection and prognosis supports a dual-purpose model that can improve patient stratification and early diagnosis before clinical manifestations. Serving as reliable molecular indicators, lncRNAs show promise as non-invasive liquid biopsy tests that not only confirm GBM but also inform clinicians about disease progression (178).

## 7 Role of lncRNAs in modulating the immunogenic profile of GBM

LncRNAs have gained attention not only as drivers of proliferation and therapy resistance but also as regulators of the GBM immune microenvironment, a dense, myeloid-dominated, and strongly immunosuppressive compartment. This section first surveys the computational signatures that use lncRNA expression to classify the GBM immune landscape and stratify patients, then turns to the mechanisms by which individual transcripts act on the principal immune populations: macrophage and microglial polarization, T-cell dysfunction, recruitment of suppressive myeloid cells, and immune checkpoint regulation. The translational consequences of immunotherapy and the strengths and limitations of the underlying evidence are also discussed. A consolidated evidence synthesis table ranked by the level of experimental support accompanies this section (**Table 5**).

**Table 5:** Current evidence supporting the role of long non-coding RNAs in immune regulation and immunotherapy-related pathways in glioblastoma.

lncRNA(s)	Study (Ref)	Primary methodology	Proposed immune mechanism	Immune cells implicated	Clinical relevance	Major limitation	Level of evidence
Functional validation in GBM							
*NEAT1*	Yi et al. (179)	Computational: TCGA correlation. Experimental: RIP-Seq, RIP, ChIP, qRT-PCR in primary GBM lines; xenograft	*PTRF*/Cavin-1 stabilizes *NEAT1*, suppressing UBXN1 and activating NF-κB to drive PD-L1 transcription and T-cell suppression	CD8+ T cells	Candidate immune-checkpoint target	No isogenic *in vivo* rescue of the *NEAT1*–PD-L1 arm	A
*NEAT1*	Toker et al. (180)	Computational: lncRNA profiling across two GBM and one melanoma ICB cohort; scRNA-seq. Experimental: *NEAT1* silencing in macrophages	*NEAT1* induced downstream of IFNγ; silencing suppresses M1 macrophage polarization and TNFα output	Macrophages, microglia, CD8+ T cells	Candidate biomarker of ICB response	Response association correlative across small cohorts	A
*H19*	Chen et al. (181)	Experimental: scRNA-seq (17 GBM); promoter ChIP; validation in 40 paired samples; circRNA vaccine in C57BL/6	*H19*-encoded micropeptide (H19-IRP) activates CCL2 and Galectin-9 transcription, recruiting MDSCs and TAMs and driving T-cell exhaustion	MDSCs, TAMs, CD8+ T cells	Candidate therapeutic target; vaccine antigen	Protein-coding and ceRNA mechanisms not fully separated	A
*LINC01018*	Hu et al. (182)	Experimental: 78 GBM tissues by flow cytometry; co-IP; CreLox EV-transfer reporter; orthotopic C57BL/6 model	*LINC01018*/miR-182-5p/Rab27B axis controls PD-L1 sorting into tumor EVs, driving microglial M2 polarization and CD8+ T-cell dysfunction (TIM-3↑, IFN-γ↓)	Microglia, macrophages, CD8+ T cells	Candidate target for ICB combination	Tumor-suppressive direction inferred from overexpression	A
*INCR1*	Saini et al. (183)	Experimental: RNAscope in 5 GBM patients pre/post IL12 therapy; ASO knockdown; RNA-seq; 3D cytotoxicity assays	*INCR1* (transcribed from *CD274* locus) binds HNRNPH1 to co-regulate PD-L1, CTLA-4, IDO1; silencing suppresses multiple checkpoints at once	T cells, Tregs	Candidate combination target with IL12 gene therapy	Small patient cohort; no *in vivo* survival data	A
*FAM131B-AS2*	Wang et al. (184)	Experimental: 149 primary GBM by qPCR; RNA pull-down, co-IP, mass spectrometry; xenograft	*FAM131B-AS2* stabilizes RPA1 via USP7, restraining the cGAS-STING pathway; inhibition raises CD8+ T-cell infiltration and ICI response	CD8+ T cells, lymphocytes	Candidate biomarker and combination target	Immune phenotype secondary to replication-stress role	A
*MIR222HG*	Fan et al. (185)	Experimental: RNA-seq of MES/PN GBM; RNA pull-down, mass spectrometry, RIP, ChIP, luciferase, Co-IP	Exosomal miR-221/222 from *MIR222HG* target SOCS3 in macrophages, releasing JAK/STAT and driving immunosuppressive polarization	Macrophages	Candidate target linking MES transition to immunosuppression	Immune arm rests on miR-221/222, not the lncRNA backbone	A
*PVT1*	Huang et al. (186)	Computational: Glioma correlation. Experimental: nuclear *PVT1* knockdown, DHX9 RIP, ChIP, *in vivo* growth	Nuclear *PVT1* binds DHX9 at the STAT1 and CX3CL1 promoters, suppressing ISGs and driving macrophage recruitment and M2 polarization	Macrophages	Candidate therapeutic target	No direct CX3CL1 manipulation to confirm recruitment	A
TMZ-associated lncRNA (*lnc-TALC*)	Li et al. (187)	Experimental: exosome isolation; ENO1 binding; murine model; C5a-targeted therapy	Tumor exosomal lncRNA binds ENO1 to activate p38 MAPK in microglia, driving M2 polarization and complement C5/C5a secretion supporting chemoresistance	Microglia, macrophages	Candidate target to reverse TMZ resistance	Immune readout centered on a single complement axis	A
*CD109-AS1*, *LINC02447*	Xu et al. (188)	Computational: methylome clustering (n=551). Experimental: siRNA knockdown and overexpression in U251/U87; Western blot; anti-PD1 cohort	Knockdown reduces PD-L1, CTLA4, FOXP3 and rescues MHC-I; both decreased in anti-PD1 responders	T cells, Tregs, NK cells	Candidate predictor of checkpoint-inhibitor response	No *in vivo* validation; small retrospective response cohort	A
Experimental expression validation							
*H19*, *SOX21-AS1*,*AC006213.5* (+3 irlncRNAs)	Yu et al. (189)	Computational: TCGA survival modeling (n=168); GSEA; scRNA-seq (4 GBM, 1,190 cells). Experimental: qRT-PCR and Western blot of genes	Prognostic irlncRNA signature; *H19* proposed via the miR-193a-3p/PLAU axis with enrichment of JAK/STAT, apoptosis, T-cell-receptor signaling	MSCs, astrocytes (paracrine, M2-skewing)	Immune-related molecular subtype classification	Immune mechanisms remain computationally inferred	B
Predominantly computational studies							
*USP30-AS1*, *HCP5*, *PSMB8-AS1*, *AL133264.2*, *LINC01684*, *LINC01506*	Gao et al. (190)	Computational: TCGA ssGSEA (n=144), mRMR, random-forest classifier. Experimental: qRT-PCR of expression only (10 GBM vs 5 non-tumor)	Co-expression links the signature with antigen presentation, NK-cell cytotoxicity, interleukin signaling; classifier AUC 0.928	B, CD4+/CD8+ T cells, neutrophils, macrophages, DCs	Immune-subtype classification and prognostic stratification	No functional validation; immune annotations from enrichment	C
Four immune-related lncRNAs (including *AC002401.4*)	Luan et al. (191)	Computational: TCGA + TCIA radiogenomics (n=57); CIBERSORT	Predicted association with macrophage polarization (M0↑, M1↓), NK-cell activation, checkpoint signaling (PD-L1 r=−0.61, CTLA-4 r=−0.43)	M0/M1 macrophages, monocytes, NK cells	Integrated radiogenomic prognostic model	Small cohort; immune estimates algorithmic	C
*AC005013.5*, *UBE2R2-AS1*,*ENTPD1-AS1*, *RP11-89C21.2*, *AC073115.6*, *XLOC_004803*	Zhou et al. (192)	Computational: TCGA microarray (n=419 training, 219 testing); Cox modeling; 931 co-expressed genes; GO/KEGG	Enrichment implicates innate and adaptive immunity, antigen presentation, Toll-like receptor and NOD-like receptor signaling; *ENTPD1-AS1* antisense to CD39	Not resolved at cell level	Prognostic risk-prediction model	In silico only; authors note validation required	C
Pyroptosis-related lncRNA signature	Xing et al. (193)	Computational: TCGA + CGGA (n=517); WGCNA, LASSO	High-risk signature predicts greater immune infiltration, higher checkpoint gene expression, better immunotherapy response but worse chemotherapy response	Mixed infiltrate (deconvolution)	Risk-stratification and immunotherapy-response model	Signature correlative; pyroptosis link inferred	C
*AL161785.1*, *LINC02611*, *PCED1B-AS1* (→ *MS4A6A*)	Bazrgar et al. (194)	Computational: TCGA + GEO ceRNA network; PPI; survival validation	Three lncRNA–miRNA axes converge on MS4A6A, a myeloid hub correlated with TAM infiltration and immune-suppressive programs	TAMs (myeloid)	Candidate prognostic biomarkers	Network inference only; no experimental validation	C
Mechanistic evidence from non-GBM immune models							
*lincRNA-Cox2*, *THRIL*, *NEAT1*, *NRON*, *NeST*, *lnc-DC*	Atianand &Fitzgerald (195)	Evidence source: mechanistic studies in murine and human immune cells (review)	Established control of macrophage activation (*lincRNA-Cox2*/NF-κB), IFN-γ induction (*NeST*/WDR5), NFAT/IL-2 signaling (*NRON*)	Macrophages, monocytes, CD8+/CD4+ T cells, DCs	Mechanistic framework for immune regulation	Not demonstrated in GBM	A†,
Evidence summarized from previous reviews							
*CASC2c*, *MALAT1*, *SNHG1*, *RP11-571M6.8*, GBM TAM lncRNAs	Varier et al. (196)	Evidence source: cancer immunotherapy review citing GBM functional and primary-tissue data	Regulation of macrophage polarization, checkpoint signaling, T-cell exhaustion; *CASC2c* abrogates M2 polarization in GBM	Macrophages, CD8+ T cells, Tregs, MDSCs	Candidate immunotherapeutic targets	Evidence varies considerably among individual lncRNAs	B

Evidence grading is presented in **Table 6**. A grade of A denotes mechanistic depth within an individual study and not independent reproducibility across studies. †The study by Atianand and Fitzgerald provides mechanistic evidence in immune cells but not in glioblastoma models; its grade (A†) reflects functional rigor in non-GBM systems.

ASO, antisense oligonucleotide; AUC, area under the curve; CGGA, Chinese Glioma Genome Atlas; ChIP, chromatin immunoprecipitation; CIBERSORT, Cell-type Identification by Estimating Relative Subsets of RNA Transcripts; co-IP, co-immunoprecipitation; CTLA-4, cytotoxic T-lymphocyte-associated protein 4; DC, dendritic cell; EV, extracellular vesicle; GBM, glioblastoma; GO, Gene Ontology; GSEA, Gene Set Enrichment Analysis; ICB, immune checkpoint blockade; ICI, immune checkpoint inhibitor; IDO1, indoleamine 2,3-dioxygenase 1; IFN, interferon; IL12, interleukin 12; ISG, interferon-stimulated gene; KEGG, Kyoto Encyclopedia of Genes and Genomes; LASSO, least absolute shrinkage and selection operator; MDSC, myeloid-derived suppressor cell; MES, mesenchymal; MHC-I, major histocompatibility complex class I; mRMR, minimum redundancy maximum relevance; NK, natural killer; PD-L1, programmed death-ligand 1; PN, proneural; PPI, protein–protein interaction; qRT-PCR, quantitative reverse-transcription polymerase chain reaction; RIP, RNA immunoprecipitation; scRNA-seq, single-cell RNA sequencing; ssGSEA, single-sample gene set enrichment analysis; TAM, tumor-associated macrophage; TCGA, The Cancer Genome Atlas; TCIA, The Cancer Imaging Archive; TIM-3, T-cell immunoglobulin and mucin-domain containing-3; TMZ, temozolomide; Treg, regulatory T cell; WGCNA, weighted gene co-expression network analysis.

To facilitate the critical appraisal of the available literature, studies were organized by the highest level of experimental evidence supporting each lncRNA’s immune-related functions, rather than by publication date, thereby emphasizing the relative strength of the evidence.

### 7.1 Immunophenotype- and prognosis-related lncRNA signatures

First, lncRNAs could not be effectors but rather readouts, with their expression used to classify the GBM immune microenvironment and stratify patients by outcome. Immunophenotype-related lncRNAs (im-lncRNAs) mark the immune subtypes of GBM and correlate with the level of tumor immune cell infiltration and have been proposed as molecular indicators of tumor immunoreactivity (195, 196). Their contribution to immune heterogeneity and clinical outcomes remains incompletely characterized until a series of cohort studies directly addressed it. These studies share a common design: bulk transcriptomes from public cohorts, immune cell estimates from deconvolution algorithms, and a multi-lncRNA index selected to predict survival or immune phenotype. They established that lncRNA expression closely tracks the tumor’s immune state; however, with one exception, they stopped at correlation and did not test how any individual transcript acts on immune cells. Therefore, they are best read as a descriptive map of the field, with the mechanistic work in the following sections, supplying the biology that these maps imply.

The clearest statement of this approach is the immunophenotype classifier in the study by Gao et al., which implemented a random forest model with minimum redundancy maximum relevance feature selection to derive six im-lncRNAs (*USP30-AS1*, *HCP5*, *PSMB8-AS1*, *AL133264.2*, *LINC01684*, and *LINC01506*) and a composite im-lncScore obtained by normalized principal component analysis (PCA) of their expression (190). The signature separated immune-high from immune-low tumors and correlated with infiltration estimates across B cells, CD4+ and CD8+ T cells, neutrophils, macrophages, and dendritic cells. However, its immune annotations rest on co-expression enrichment rather than functional assays, and the only experimental step was confirmation of expression by qRT-PCR. Zhou et al. applied the same logic to prognosis, screening genome-wide lncRNA profiles in 419 TCGA patients and validating a six-lncRNA risk model (*AC005013.5*, *UBE2R2-AS1*, *ENTPD1-AS1*, *RP11-89C21.2*, *AC073115.6*, and *XLOC_004803*) in a further 219 patients (192). Pathway enrichment of co-expressed genes implicated innate and adaptive immunity, antigen presentation, and Toll-like and NOD-like receptor signaling. Notably, *ENTPD1-AS1* is antisense to *ENTPD1* (CD39), the ectoenzyme that drives the adenosine pathway in regulatory T cells, which makes it an attractive candidate, even though that link has never been tested (192).

Three further studies added resolution without changing the level of evidence. Xu et al. defined an immune subtype of glioma from the lncRNA methylome, a layer that captures the crosstalk among DNA methylation, lncRNA expression, and immune regulation, and, unusually for this group, moved beyond correlation: their two markers, *CD109-AS1* and *LINC02447*, were validated by knockdown and overexpression in GBM cells and were reduced in anti-PD1 responders (188). Because that work establishes a checkpoint mechanism rather than a signature alone, it is the only member of this computational set that provides a functional readout; the mechanism is detailed in later sections of this article. Luan et al. integrated four immune-related lncRNAs with two radiomic features from paired TCGA and TCIA data, producing a model that stratified survival by both risk score and Kaplan-Meier analysis. Furthermore, CIBERSORT linked high-risk patients to a shift from M1 toward M0 macrophages (191). Yu et al. screened 168 GBM transcriptomes, identified 17 prognostic immune-related lncRNAs, and derived a six-lncRNA signature from them that resolved 4 molecular subtypes (189). The favorable subtype (subtype A) carried *IDH1*, *ATRX*, and *EGFR* mutations and low expression of four high-risk lncRNAs (*H19*, *HOTAIRM1*, *AGAP2-AS1*, and *AC002456.1*) together with low *KRT8*, whereas poor-survival subtypes were reported to have infiltrations by mesenchymal stem cells and astrocytes, cell types that supply the M2-skewing paracrine signals and were more sensitive to gefitinib. Three hub genes from this model, *KRT8*, *NGFR*, and *TCEA3*, were validated as candidate oncogenes, with *KRT8* proposed to act through T-cell receptor, apoptotic, or JAK/STAT signaling. The same study proposed *H19* as a ceRNA that acts on *PLAU* via miR-193a-3p (189). Collectively, the six lncRNA panels reported by Gao, Zhou, and Yu share no transcripts in common. The wider set of computational models in this area, including the pyroptosis-related signature of Xing et al. and the ceRNA network of Bazrgar et al. that converges on the myeloid marker *MS4A6A*, likewise draws on largely non-overlapping gene lists built from the same few public cohorts (193, 194). The fact that immune-related lncRNA signatures can be derived from the same data without converging on a shared core is itself a finding: it points to a statistical space rich enough to yield numerous predictive models and to the absence of a validated mechanistic anchor that would explain why any particular panel should be preferred.

### 7.2 LncRNAs and macrophage and microglia polarization

Tumor-associated macrophages (TAMs) and microglia comprise a large fraction of the glioblastoma mass, with many tumors approaching a third to a half of all cells, and their functional state sets the immunological tone of the lesion. The bulk of this compartment is closer to an M2-like immunosuppressive phenotype than to the M1-like state associated with antitumor activity. Cytokines abundant in the GBM microenvironment, including TGF-β (transforming growth factor-beta), IL-10, IL-4, and CSF-1 (colony-stimulating factor 1), push macrophages toward the M2-like program, while JAK1/2-STAT1 and NF-κB signaling that supports M1 activation is actively held in check (197). A growing set of lncRNAs operates at these control points and has been investigated using direct functional experiments.

The clearest GBM-specific example of a lncRNA that restrains the M2 program is *CASC2c*, which abrogates M2 polarization and reduces tumor growth (196). Varier et al. identified distinct lncRNA profiles in M1 versus M2 tumor-infiltrating macrophages in primary GBM tissues, establishing that the polarization axis in GBM is under lncRNA control rather than merely correlated with it. Until recently, the field lacked mechanisms at the level of specific transcripts.

*NEAT1* sits where interferon signaling and macrophage states meet. Toker et al. found that *NEAT1* is induced downstream of IFN-γ and that its silencing suppresses M1 macrophage polarization and reduces TNF-α and other inflammatory cytokines (180). Single-cell sequencing identified *NEAT1* in tumor cells, macrophages, and T cells, with high tumor-cell *NEAT1* levels correlating with denser macrophage and microglial infiltration (180). It is worth noting that *NEAT1* here supports the inflammatory M1 arm, which could explain its high expression being associated with better checkpoint-blockade responses (180). The same transcript also feeds into the immunosuppressive circuit. Yi et al. showed that the RNA-binding protein PTRF/Cavin-1 stabilizes *NEAT1*, suppresses UBXN1 (a negative regulator of NF-κB signaling), and activates NF-κB to drive PD-L1 transcription (179). *NEAT1* thus sits on both sides of the ledger, depending on which protein partner and cell type dominate, reminding us that a single lncRNA does not carry a fixed immunological sign.

The second mechanism involves the routing of polarization through extracellular vesicles. Hu et al. described the *LINC01018*/miR-182-5p/Rab27B axis, in which low Rab27B levels lead to greater PD-L1 loading into tumor-derived vesicles. Then, microglia that take up these vesicles upregulate PD-L1 and shift toward a CD206+ M2 state (182). In 78 GBM tissues, low Rab27B levels were associated with increased macrophage and monocyte infiltration. An orthotopic model confirmed that disrupting the axis reduced TAMs and improved anti-PD-L1 efficacy. A conceptually similar route was reported by Li et al., in which a temozolomide-associated tumor exosomal lncRNA binds to ENO1 in recipient microglia, activates p38 MAPK, drives M2 polarization, and elicits complement C5/C5a secretion, thereby supporting chemoresistance (187). Therefore, identifying exosomal lncRNA transfer as a mechanism of polarization strengthens the concept that this is a real feature of GBM biology, rather than a cell-line artifact.

Transcriptional and chromatin-level control is represented by *PVT1* and *MIR222HG*, respectively. Huang et al. showed that nuclear *PVT1* binds to DHX9 and occupies the STAT1 and CX_3_CL1 promoters, suppressing interferon-stimulated genes while raising CX_3_CL1 to recruit macrophages and promote M2 polarization (186). Fan et al. linked the proneural-to-mesenchymal transition of glioma stem cells to immunosuppression, where cotranscribed miR-221 and miR-222 from the *MIR222HG* locus are delivered to macrophages via exosomes, where they target SOCS3 and promote JAK/STAT-driven polarization (185). Both studies used RNA pull-down, RIP (RNA immunoprecipitation), ChIP (chromatin immunoprecipitation), and mass spectrometry; therefore, the mechanistic claims rest on direct binding data rather than co-expression.

Two computational studies fill in the population-level picture without claiming a mechanism. Luan et al. found by CIBERSORT that high-risk tumors carried more M0 and fewer M1 macrophages, with checkpoint genes inversely correlated to the immune-related lncRNA score (191). On the other hand, Yu et al. reported that poor-prognosis subtypes were enriched for mesenchymal stem cells and astrocytes, cell types that supply M2-skewing paracrine signals (189). Network analysis by Bazrgar et al. added that several lncRNA-miRNA axes converge on *MS4A6A*, a myeloid marker associated with TAM infiltration (194). These studies are considered at evidence level C, but they converge. Independent pipelines consistently identify macrophage composition as the immune feature most tightly linked to the lncRNA-defined risk.

### 7.3 LncRNAs, T-cell dysfunction, and recruitment of suppressive myeloid populations

If the macrophage axis sets the background tone of the GBM microenvironment, T-cell dysfunction ultimately blunts the anti-tumor response, and several lncRNAs act directly on it. The strongest single result comes from the study Chen et al., which reported that *H19* encodes a 256-amino-acid micropeptide, H19-IRP, which is functionally separate from the RNA transcript (181). H19-IRP binds to the CCL2 and Galectin-9 promoters and activates their transcription. CCL2 recruits MDSCs (myeloid-derived suppressor cells) and TAMs, while Galectin-9 induces T-cell exhaustion. Single-cell sequencing of 17 GBM samples revealed that *H19* expression in tumor cells was negatively correlated with activated CD8+ T cells and positively correlated with MDSCs, macrophages, and Tregs. This revises how *H19* should be discussed. The earlier signature work of Yu et al. framed *H19* as a ceRNA acting through the miR-193a-3p/*PLAU* axis (189). This mechanism is not incorrect, but the protein-coding route now carries stronger direct evidence for immune function. The two are not mutually exclusive and likely operate in parallel.

A distinct mechanism links DNA replication stress and T-cell exclusion. Wang et al. showed that *FAM131B-AS2*, located in the frequently amplified 7q34 region, stabilizes RPA1 (Replication Protein A1) through USP7 and activates ATR signaling to protect single-stranded DNA (184). Its overexpression inhibited CD8+ T-cell infiltration, and its inhibition activated the cGAS-STING pathway, increasing lymphocyte infiltration and improving the response to checkpoint inhibitors in a cohort of 149 primary tumors. This is the only study that connects replication stress sensing to lncRNA-driven immune exclusion in GBM, a distinct mechanism from the ceRNA and checkpoint pathways that dominate the rest of the literature.

As previously discussed, the extracellular vesicle axis also induces a measurable T-cell phenotype. In the study by Hu et al., vesicle-delivered PD-L1 was accompanied in patient tissues by increased TIM-3 and reduced IFN-γ expression on CD8+ T cells, a signature of functional exhaustion, even though absolute CD8+ T cell numbers did not change (182). This indicates that lncRNA does not necessarily exclude T cells as much as it disarms the ones that are present.

Atianand and Fitzgerald cataloged lncRNAs that govern T-cell programs in non-tumor settings, including *NRON*, which sequesters NFAT to restrain IL-2 and T-cell activation, and *NeST*, which deposits H3K4me3 at the IFN-γ locus through WDR5 in CD8+ T cells (195). Varier et al. compiled candidate T-cell and Treg mechanisms in glioma, including the inverse relationship between *RP11-571M6.8* and PD-1, PD-L1, and CTLA4 (196). These are biologically established and plausible in GBM, but they have not been demonstrated yet.

### 7.4 LncRNAs and immune-checkpoint regulation

Checkpoint regulation is where the GBM lncRNA literature is most mature because several transcripts have been directly associated with PD-L1 and broader checkpoint programs with functional experiments. The CD109-AS1 and LINC02447 pair, characterized by Xu et al., is the best example (188). Knockdown in U251 and U87 cells reduced PD-L1, CTLA4, and FOXP3 protein and rescued MHC-I, while overexpression had the opposite effect. Additionally, both transcripts were lower in patients who responded to anti-PD1 therapy. This is the only checkpoint mechanism in the dataset validated by both loss- and gain-of-function in GBM cells with protein-level readouts.

*NEAT1* provides a parallel and mechanistically detailed route for PD-L1. Yi et al. traced the full axis: PTRF/Cavin-1 (polymerase I and transcript release factor/Caveolae-associated protein 1) stabilizes *NEAT1*, which suppresses UBXN1. Loss of UBXN1 activates NF-κB, which in turn transactivates PD-L1, as confirmed by RIP-Seq, RIP, and ChIP (179). The impact of two different lncRNAs, *NEAT1* and *HOTAIR* in an earlier study, on the UBXN1/NF-κB is one of the more striking patterns in the literature.

The most therapeutically provocative checkpoint finding concerns *INCR1*. Saini et al. showed that *INCR1*, transcribed from the PD-L1 (*CD274*) locus and co-induced with PD-L1 by IFN-γ, binds to HNRNPH1 (heterogeneous nuclear ribonucleoprotein H1) and regulates a panel of immunosuppressive genes, including PD-L1, CTLA-4, and IDO1 (183). Knocking down *INCR1* showed greater suppression of these markers than silencing PD-L1 alone, and this was observed in patient-derived cells using antisense oligonucleotides. The implication is structural rather than incremental: a single lncRNA target can reach a wider slice of the immune-evasion program than a single checkpoint antibody, which reframes the meaning of targeting a checkpoint in GBM. The IDO1 arm also ties back to Treg biology through the kynurenine pathway, linking this section to the suppressive cell recruitment described earlier.

Supporting evidence comes from computational literature and should be considered accordingly. Varier et al. compiled additional checkpoint-linked transcripts, including the *MALAT1*/miR-195/PD-L1 axis and the inverse correlation of *RP11-571M6.8* with multiple checkpoints (196). Radiogenomic analysis by Luan et al. reported negative correlations between an immune-related lncRNA score and both PD-L1 and CTLA-4 expression (191). These are consistent with the functional studies, but cannot stand in for them.

### 7.5 Implications for immunotherapy response

The therapeutic question that matters for GBM is not whether lncRNAs correlate with prognosis but whether they can be manipulated to improve response to immunotherapy. Three lines of evidence are directly related to this. The first is prediction. Toker et al. found that *NEAT1* was upregulated in patients with GBM with longer survival after anti-PD-1/PD-L1 treatment across two independent cohorts, with a similar pattern in melanoma (180). The proposed logic connects IFN-γ in the microenvironment to *NEAT1* induction in tumor and myeloid cells, and then to M1-like activation and a more inflamed, treatment-responsive tumor.

The second line is interventional. Saini et al. addressed a real clinical failure: IL12 gene therapy in GBM provokes compensatory upregulation of PD-L1 and *INCR1*, and adding PD-1/PD-L1 antibodies did not restore efficacy (183). Silencing *INCR1* suppressed a broader immunosuppressive program and outperformed checkpoint antibodies in 3D killing assays using tissue from patients before and after IL12 therapy. The third line is sensitization through innate sensing. Wang et al. showed that inhibiting *FAM131B-AS2* activated cGAS-STING, increased lymphocyte infiltration, and improved checkpoint-inhibitor response, positioning it as both a prognostic indicator and a candidate combination target (184). Two further studies provide supporting evidence: a circular RNA vaccine against the H19-IRP micropeptide triggered cytotoxic T-cell responses and slowed GBM growth *in vivo*, thereby converting a suppressive lncRNA product into a vaccine antigen (181). Hu et al. showed that disrupting the *LINC01018*/Rab27B vesicle axis enhanced anti-PD-L1 efficacy, with vesicle re-injection reversing the benefit, which closes the causal loop between mechanism and therapeutic effect (182).

Computational signature work also identified a pyroptosis-related lncRNA signature in which high-risk patients showed greater immune infiltration, higher checkpoint expression, and predicted better immunotherapy response but worse chemotherapy response (193). The pyroptosis framework is mechanistically attractive because pyroptotic death releases danger signals that can prime cGAS-STING and T-cell recruitment, linking this signature conceptually to the *FAM131B-AS2* finding. However, the link is inferred from transcriptomic correlation, and the prediction is a model output rather than a measured clinical response. Collectively, these studies move the field from lncRNAs correlating with immune phenotype toward specific lncRNAs that can be targeted to alter immunotherapy outcomes, and that shift is the strongest argument for giving this class of molecules a place in GBM immunotherapy strategy rather than only in prognostic models.

### 7.6 Critical appraisal and limitations of immunophenotype-related lncRNAs

**Figure 5:**
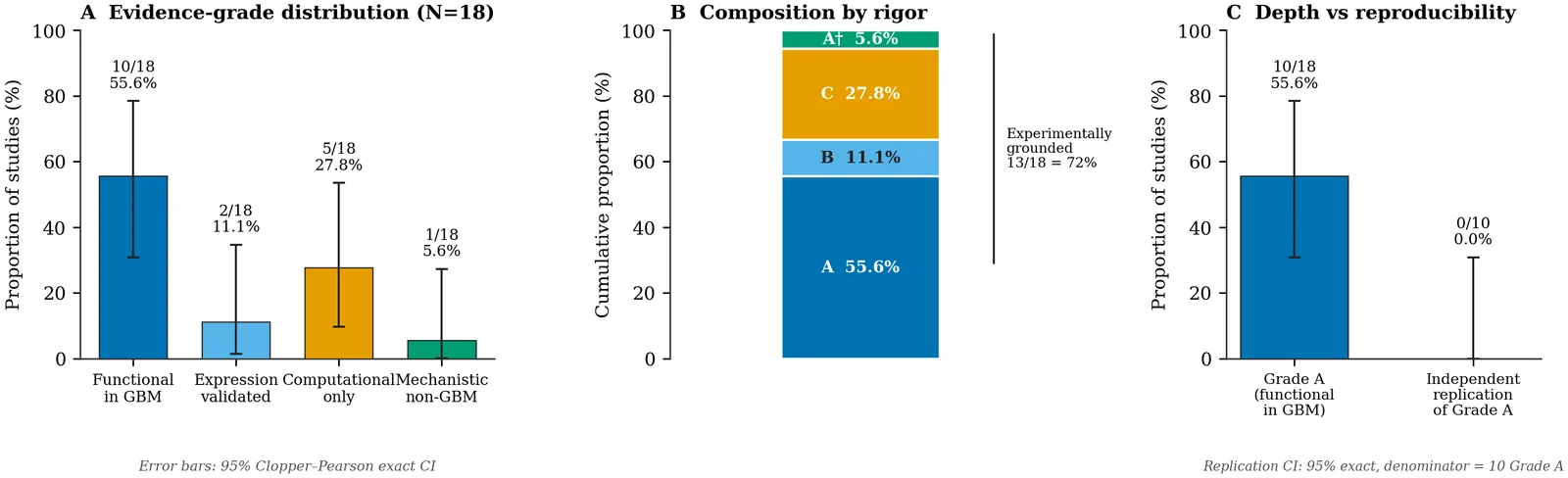
The quantitative synthesis of the 18 selected studies. **(A)** Proportion of studies in each evidence grade, with 95% Clopper-Pearson exact confidence intervals; counts are above each bar. **(B)** Cumulative composition by rigor; the bracket marks the experimentally grounded fraction (Grade A, B, or A†; 13/18, 72%). **(C)** The contrast between mechanistic depth and reproducibility: 55.6% of studies reached Grade A functional validation in GBM, but none of those ten Grade A studies were independently replicated (0/10). Grades: A, functional validation in GBM; B, expression validated, mechanism inferred; C, computational only; A†, mechanistic evidence in non-GBM immune cells. The color palette is colorblind-safe.

To critically evaluate the current evidence, 18 included immunophenotype-related lncRNA studies were classified by the highest level of experimental support for their immune-related conclusions, ranging from direct functional validation in glioblastoma to computational associations alone. To complement this qualitative framework, the distribution of evidence grades was summarized using exact confidence intervals. The grading criteria, study-level evidence synthesis, and overall distribution are presented in **Tables 5**, **6** and **Figure 5**. The following appraisal integrates these findings to assess the methodological strengths, limitations, and overall maturity of the current evidence.

**Table 6:** The evidence hierarchy used to grade the immune-related functions of long non-coding RNAs in GBM, with the distribution of the 18 selected studies.

Level	Definition	Typical study design	Number of studies	Proportion (95% CI)
A	Functional validation in GBM models demonstrated a direct mechanistic immune-related effect (for example, lncRNA knockdown or overexpression producing a measured change in immune-cell phenotype, checkpoint protein expression, or *in vivo* immune response).	Knockdown or overexpression with protein-level or *in vivo* readouts	10	55.6% (30.8–78.5)
B	Experimental validation of lncRNA expression in clinical specimens or GBM tissues (e.g., by qRT-PCR or western blotting), with the immune mechanism inferred computationally rather than directly tested.	qRT-PCR or Western blot of tissue expression; mechanism inferred	2	11.1% (1.4–34.7)
C	Computational evidence only, including transcriptomic correlation, immune deconvolution, pathway enrichment, or survival modeling, without experimental validation of the proposed immune function.	TCGA/CGGA signature modeling; CIBERSORT or ssGSEA; ceRNA networks	5	27.8% (9.7–53.5)
A†	The functional mechanism established in non-GBM immune cells provides a mechanistic framework that has not yet been demonstrated in glioblastoma.	Mechanistic studies in murine and human immune cells (review)	1	5.6% (0.1–27.3)

Proportions of the 18 studies, with 95% Clopper-Pearson exact confidence intervals. A Grade A reflects mechanistic depth within a single study and does not denote independent replication. Among the ten Grade A studies, none have been independently replicated in GBM (0/10, 95% CI 0.0–30.8). † Mechanism established in non-GBM immune cells.

However, the apparent consistency of the current evidence should be interpreted cautiously because several methodological limitations remain. First, the computational signatures are based on a narrow data foundation. The five signature studies discussed in Section 7.1, together with the pyroptosis and ceRNA network models, repeatedly draw on the same TCGA and CGGA cohorts, and their immune-cell estimates come from deconvolution algorithms rather than direct measurements. Repeated interrogation of the same discovery cohorts creates an impression of reproducibility without contributing independent biological evidence. Second, signature proliferation is a warning. The six-lncRNA panels of Gao, Zhou, and Yu share no transcripts, and the broader set of immune-related lncRNA models converges on no common core. This points to a feature space rich enough to generate many predictive models, few of which have been tested against each other or in prospective cohort studies.

Third, the mechanistic hierarchy is uneven. Among the functionally validated studies, *CD109-AS1*/*LINC02447* (188) and the mechanistic papers (179–187) provide direct loss- or gain-of-function evidence in GBM systems, whereas most signature-level immune annotations are co-expression inferences or ceRNA hypotheses that have not been confirmed with RNA-protein binding, reporter, or immune-cell functional assays. The grading scheme in the table reflects this asymmetry. Several Grade A mechanisms also place the immune phenotype downstream of a primary non-immune function, as with *FAM131B-AS2* (replication stress) and the opposing roles reported for *NEAT1*, so the apparent maturity of the mechanistic column should be read with that caveat in mind; the reproducibility point is quantified in **Table 6** and **Figure 5**. The radiogenomic model of Luan et al. is further constrained by a small cohort and by imaging parameter heterogeneity that was not fully controlled, which limits the reliability of its imaging-derived risk groups (191).

Finally, the immunotherapy-response data, while the most clinically interesting, derive from small and largely retrospective cohorts, and several depend on response-prediction scores rather than measured clinical endpoints. The interventional results for *INCR1* and *FAM131B-AS2* are mechanistically strong but remain preclinical. These limitations do not diminish the case for lncRNAs as immune regulators in GBM; they define the experiments, independent replication, prospective validation, and immune-cell-resolved functional testing that would let the field convert a promising map into actionable targets.

At first glance, the predominance of Grade A studies (55.6%) could be interpreted as evidence that the field is supported by extensive mechanistic validation. A closer examination, however, leads to a different conclusion. Three quantitative observations derived from **Table 6** and **Figure 5** illustrate why. First, the confidence intervals are wide: with only eighteen studies, the estimated proportion of Grade A evidence is compatible with values ranging from approximately one-third to four-fifths of the literature, indicating considerable uncertainty around the point estimate. Second, collapsing the grades into experimental-grounding (A, B, or A†) versus computational-only (C) yields 13 of 18 studies (72.2%, 95% CI 46.5–90.3). However, an exact binomial test against an equal distribution was not statistically significant (p = 0.096), indicating that the apparent dominance of experimentally grounded work does not provide sufficient statistical evidence that experimentally supported studies predominate over computational studies at the current sample size. Third, and most important, independent replication among the ten Grade A studies is zero (0/10, 95% CI 0.0–30.8): every mechanistic claim rests on a single group, cohort, and model system. Depth of evidence within studies is therefore high, while reproducibility across studies is unestablished, and the two must be reported together to avoid overstating maturity.

Overall, the available evidence supports the biological relevance of immune-related lncRNAs in glioblastoma, but it remains insufficient to establish causal regulatory networks for most candidate molecules. Consistent with both the quantitative analyses and the qualitative assessment presented in this review, future work should prioritize independent replication of mechanistic findings, prospective validation of predictive biomarkers, and immune cell-resolved functional studies to establish the causal roles of lncRNAs that are currently supported primarily by statistical associations.

## 8 Mechanisms of lncRNAs delivery to the brain and their limitations

The optimal nanoparticle design for transporting therapeutic lncRNAs across the BBB must account for the specific requirements for RNA stabilization and release, as well as the structural and functional challenges posed by this selective barrier (198). To ensure that the nanoparticle can pass through the small gaps between BBB cells without triggering immune clearance, it must first be engineered to a size typically between 10 and 100 nanometers (199). Another factor is shape; spherical or slightly elongated nanoparticles frequently exhibit the longest circulation times and the strongest interactions with BBB transport receptors (198). Additionally, the surface charge is important; a near-neutral or slightly positive charge improves BBB traversal by electrostatically interacting with the BBB’s negatively charged cellular membranes without risking rapid removal from circulation due to excessive positive charge (198). The surface of the nanoparticle can be modified with ligands or antibodies specific to receptors widely present on BBB endothelial cells, such as transferrin or insulin receptors, to enable receptor-mediated transcytosis and active targeting (198). Applying polyethylene glycol (PEG) or other hydrophilic polymers to the nanoparticle also helps “camouflage” it from the immune system, extending its circulation and increasing the likelihood of crossing the BBB (198).

The core of the nanoparticle must shield the RNA payload from enzymatic degradation in circulation and provide regulated release upon reaching target cells, thereby delivering lncRNA (198). Utilizing biocompatible and biodegradable materials, such as lipid- or polymer-based matrices that release the lncRNA in response to triggers like pH or enzymatic conditions present in GBM cells, is necessary for this (198). Additionally, for the nanoparticle to deliver sufficient lncRNA for efficient gene silencing or regulation, it must achieve high encapsulation efficiency (200). The nanoparticle should encourage cellular absorption by GBM cells once it has crossed the BBB (199). This is frequently accomplished by surface changes that attach to markers unique to GBM (199). The release of lncRNA in the acidic tumor microenvironment or upon internalization into GBM cells can be maximized by incorporating stimuli-responsive features, such as pH-sensitive bonds (200). To ensure accurate, efficient, and secure delivery to brain tumor cells, the ideal nanoparticle for lncRNA distribution across the BBB would combine size, shape, surface changes, immune evasion, targeting capabilities, and payload stability (200). However, delivering lncRNAs to target GBM in the brain poses substantial obstacles, owing to the complex anatomical and physiological barriers of the central nervous system (25).

The large size and structural complexity of lncRNAs make transport across the BBB much more problematic (26). Furthermore, the heterogeneity of GBM tumors complicates the issue, as distinct subpopulations of tumor cells may respond differently to lncRNA-based treatments, making it challenging to achieve consistent therapeutic outcomes (26). In addition, lncRNAs are susceptible to degradation by nucleases in circulation, leading to rapid clearance and reduced bioavailability at the tumor site (25). This requires the development of efficient delivery technologies that preserve lncRNAs from degradation and ensure their targeted delivery to glioma cells (201). Moreover, nanoparticles are rapidly cleared by the immune system, limiting their circulation time and reducing the likelihood of successful delivery to the brain (202). When nanoparticles reach the target region, they may become trapped in endosomes, blocking the release of lncRNAs into the cell cytoplasm or nucleus (202). Also, concerns about nanoparticle biocompatibility and toxicity persist, as certain materials may elicit immunological responses or neuroinflammation, leading to further brain injury (202). Lastly, the challenge of scaling nanoparticle manufacturing while maintaining consistency is also a barrier to clinical use (203).

A key yet often neglected delivery challenge is that lncRNAs exhibit physicochemical characteristics that are fundamentally distinct from those of conventional small-molecule chemotherapies, such as temozolomide (203). Unlike small molecules, which are compact, relatively stable compounds capable of passive membrane diffusion, lncRNAs are large polyanionic transcripts ranging from approximately 200 nucleotides to several kilobases in length (204, 205). Their massive molecular size, dense negative charge along the phosphodiester backbone, and complex tertiary folding structures collectively prevent passive diffusion across any biological membrane (203, 206). Naked RNA molecules exhibit a short circulatory half-life due to rapid degradation by endogenous serum nucleases and reticuloendothelial clearance, posing major obstacles to systemic therapeutic delivery (207). Even upon cellular uptake, lncRNAs are prone to endosomal sequestration, blocking their release into the cytoplasm or nucleus where they exert their regulatory functions (201). These lncRNA-specific physicochemical properties make the engineering of an effective delivery vehicle substantially more demanding than for conventional chemotherapy (203).

To address these challenges, nanocarriers such as liposomes, nanoparticles, or viral vectors can be engineered to cross the BBB and deliver lncRNAs directly to the tumor (203). On the other hand, even these solutions have limitations (203).

### 8.1 Different types of nanoparticles and their limitations

**Figure 6:**
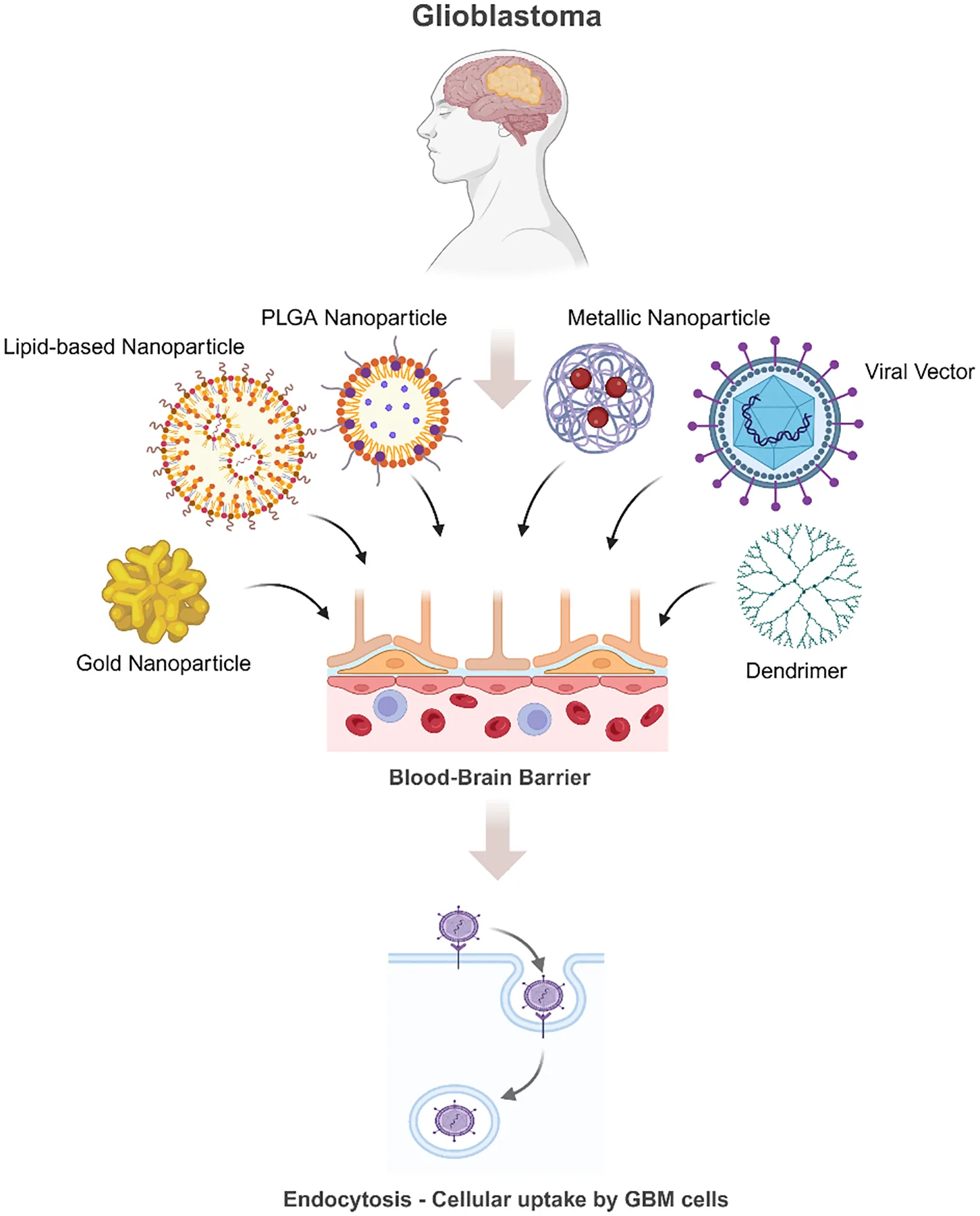
Nanoparticle-mediated delivery across the BBB for targeting GBM cells. Nanoparticles, including liposomes, polymeric nanoparticles, dendrimers, viral vectors, and other delivery systems, are designed to traverse the BBB, a selective barrier that restricts most substances from entering the brain. After crossing the BBB, the nanoparticles undergo endocytosis, enabling cellular uptake by GBM cells. This approach holds promise for delivering targeted treatments directly to GBM, potentially improving therapeutic efficacy.

Lipid-based nanoparticles, such as liposomes and solid lipid nanoparticles (SLNs), have shown promise in drug delivery systems but face several limitations (**Figure 6**) (208). Liposomes, despite their biocompatibility and ability to encapsulate both hydrophilic and hydrophobic drugs, suffer from low structural stability, which can lead to premature drug release (209). Additionally, they are rapidly cleared from circulation by the reticuloendothelial system (RES), particularly macrophages in the liver and spleen, thereby shortening their half-life and reducing therapeutic efficacy (208). SLNs, while offering greater stability than liposomes, often exhibit drug expulsion during storage and have limited penetration across the BBB, making it difficult for these systems to deliver therapeutic agents to the brain efficiently (209).

Polymeric nanoparticles, including those made from poly (lactic-co-glycolic acid) (PLGA) and chitosan, are another popular drug-delivery vehicle but also come with their own set of challenges (210). PLGA nanoparticles degrade slowly, which can result in prolonged retention of the delivery vehicle in the body, potentially leading to unwanted accumulation in non-target tissues (211). Additionally, acidic by-products of PLGA degradation can trigger local inflammatory responses and adversely affect drug-release kinetics (211). Chitosan nanoparticles, known for their biocompatibility and mucoadhesive properties, can elicit immune responses and exhibit inconsistent drug-release profiles, often releasing drugs either too slowly or too rapidly, thereby limiting their effectiveness (212).

Dendrimers, highly branched, nanoscale polymers, are advantageous due to their precise structure and high drug-loading capacity (213). However, their highly functional surface groups, which enable multivalent drug conjugation, can lead to toxicity, particularly when cationic dendrimers interact with negatively charged cell membranes, thereby causing membrane disruption and cytotoxicity (213). Moreover, dendrimer synthesis is complex, requiring multiple labor-intensive steps to control size and branching, which makes scaling up production difficult and costly (213).

Gold nanoparticles (AuNPs) are widely studied for their tunable size, shape, and surface properties, offering versatility for a range of biomedical applications (214). However, their tendency to accumulate in non-target organs, particularly the liver and spleen, poses a significant risk of long-term toxicity (215). Additionally, at higher doses, AuNPs can induce oxidative stress and cellular damage, further compounding their toxicity concerns (216). Their production cost is also prohibitive, as the acquisition and functionalization of AuNPs are costly, limiting their widespread clinical application (214).

Metallic nanoparticles, such as iron oxide nanoparticles, are gaining attention for their potential in magnetic targeting and hyperthermia treatments (217). However, they face significant challenges due to the shallow penetration depth of magnetic fields, which limits their effectiveness in deeper tissues and organs (218). Furthermore, iron-based magnetic nanoparticles can undergo oxidative stress, generating reactive oxygen species (ROS) that may cause cytotoxicity and interfere with normal cellular processes (218). This limits their safe application *in vivo* without additional surface modifications or antioxidant strategies (218). Together, these nanoparticle systems (**Figure 6**), while promising for various biomedical applications, face substantial hurdles in stability, toxicity, and efficient drug delivery, especially in challenging environments such as the central nervous system and non-target tissues (210).

### 8.2 Different types of viral vectors and their limitations

Viral vectors, though capable of highly efficient gene transfer, come with a variety of technical and safety challenges that complicate their use in GBM therapy, particularly for delivering lncRNAs (219). A major limitation of these vectors is the risk of immunogenicity (the ability to trigger a host immune response) (219). This can lead to rapid clearance of viral vectors from circulation, reducing their therapeutic efficacy and triggering adverse immune reactions (219). Additionally, the risk of insertional mutagenesis, in which the viral genome integrates into the host DNA at unintended sites, may disrupt essential genes and potentially induce oncogenesis (220). Viral vectors also have limited cargo capacities, which restrict the delivery of larger therapeutic molecules, including many lncRNAs that exceed their packaging limits (220).

Adeno-associated viruses (AAVs) are a popular choice for gene therapy due to their relatively low immunogenicity, high efficiency in transducing non-dividing cells, and ability to cross the BBB (221). Certain AAV serotypes, such as AAV9, are particularly adept at targeting neurons and glial cells, making them attractive for neurological diseases like GBM (222). However, AAVs have a limited cargo capacity of approximately 4.7 kb, which constrains their ability to carry larger lncRNAs or more complex regulatory elements required for precise gene modulation (223). Furthermore, pre-existing immunity to AAVs, arising from prior exposure to wild-type viruses in the human population, can reduce their therapeutic effectiveness, as neutralizing antibodies may target and eliminate the vectors before they reach the brain (224).

Lentiviral vectors (LVs) offer the advantage of integrating into the host genome, enabling long-term, stable expression of therapeutic transgenes, including larger lncRNAs (225). LVs can carry inserts up to approximately 8–10 kb, making them suitable for larger genetic elements (225). However, the ability of lentiviruses to integrate into the genome raises concerns about insertional mutagenesis, as integration at unintended genomic sites could disrupt critical genes and potentially lead to malignancies (225). Additionally, lentiviruses can elicit immune responses, particularly in immunocompromised individuals, complicating their safe use in GBM therapy (225). Adenoviruses, in contrast to AAVs and lentiviruses, have a much larger cargo capacity (up to ~36 kb), enabling delivery of larger payloads, including substantial lncRNAs, regulatory sequences, and additional therapeutic elements (226). Adenoviruses are non-integrating, which reduces the risk of insertional mutagenesis (226). However, they are highly immunogenic, provoking strong innate and adaptive immune responses that can lead to rapid vector clearance and inflammation (226). These immune reactions may limit the therapeutic window of adenoviruses, reducing their efficacy in long-term applications and increasing the risk of adverse side effects (226).

Herpes simplex virus (HSV) vectors offer among the largest cargo capacities (~150 kb), making them ideal for delivering very large genetic elements, such as full-length lncRNAs and other complex constructs (227). HSVs naturally target neurons, which makes them particularly useful for neuro-oncological applications (227). However, HSV vectors face significant hurdles due to neurotoxicity unless properly engineered to reduce virulence (227). They also elicit strong immune responses, which can lead to inflammation and rapid vector clearance, potentially negating their therapeutic benefits (227). In summary, while viral vectors offer significant advantages in the efficient delivery of lncRNAs to GBM cells, the challenges of BBB penetration, off-target effects, immune responses, and controlled gene expression must be carefully addressed to optimize their use (210). Advances in vector engineering, such as reducing immunogenicity, enhancing cell-specific targeting, and optimizing payload capacity, are essential to fully unlock the potential of viral vectors in lncRNA-based GBM therapies (210).

### 8.3 Delivery of lncRNAs therapeutics for GBM treatment

Despite these challenges, several proof-of-concept studies have demonstrated the feasibility of nanoparticle-mediated lncRNA targeting in glioblastoma. For example, MALAT1, an oncogenic lncRNA implicated in glioma progression and chemoresistance, has been successfully targeted using a receptor-directed nanocomplex carrying anti-MALAT1 siRNA (228). In preclinical glioblastoma models, nanocomplex-mediated MALAT1 silencing enhanced the antitumor efficacy of temozolomide and improved therapeutic outcomes compared with conventional treatment alone, providing evidence that nanoparticle-based delivery systems can effectively modulate lncRNA expression *in vivo* and overcome therapeutic resistance (228). This study highlights the potential of nanotechnology-enabled lncRNA-targeted therapeutics strategies for glioblastoma (228).

Another example of nanoparticle-mediated lncRNA targeting in GBM is the delivery of siRNA against the lncRNA *NONHSAT159592.1* using nanoparticle-based systems. This approach enabled the efficient silencing of *NONHSAT159592.1* in GBM cells, resulting in significant inhibition of tumor-promoting behaviors, including cell proliferation, migration, and invasion. Mechanistically, *NONHSAT159592.1* silencing suppressed the ITGA3/FAK/PI3K/AKT signaling pathway, which is involved in regulating cancer cell survival, growth, and invasive capacity (229). *In vivo*, nanoparticle-mediated delivery of si-NONHSAT159592.1 reduced glioblastoma tumor progression, highlighting the potential of nanoparticle-based lncRNA targeting as a therapeutic strategy for GBM treatment (229). Although numerous lncRNAs have been implicated in glioblastoma pathogenesis, only a limited number of studies have evaluated nanoparticle-mediated lncRNA targeting *in vivo*. Consequently, the development of delivery platforms capable of efficient BBB penetration, tumor-specific accumulation, and intracellular release remains a major challenge for the clinical translation of lncRNA-based therapeutics in GBM.

Viral vector systems in glioblastoma have mainly been used as experimental gene-silencing tools for functional investigation of lncRNAs rather than as established therapeutic delivery platforms. In this context, most available evidence relies on RNA interference-based knockdown approaches in preclinical GBM models, without translation into clinically validated viral lncRNA therapies. The oncogenic lncRNA *lncHERG* has been functionally studied in glioblastoma using knockdown approaches, where its suppression reduced glioma cell proliferation, migration, and invasion while increasing apoptosis, supporting its role in GBM malignancy (230). Importantly, although these findings demonstrate that lncRNA expression can be effectively modulated in preclinical GBM systems, current studies remain restricted to basic functional knockdown experiments rather than translational *in vivo* applications. Consequently, very limited evidence exists linking viral vectors to clinically relevant, lncRNA-targeted therapeutic delivery platforms in GBM.

## 9 Conclusion

Compared with other invasive GBM diagnostic methods, circulating genetic biomarkers are easier, faster, and more cost-effective. These biomarkers offer a promising approach to diagnosis, treatment, and prognosis, and to avoiding unnecessary chemotherapy by early identification of potential chemoresistance. LncRNAs have shown a strong correlation with GBM diagnosis, prognosis, and tumor grading, suggesting additional mechanisms underlying chemoresistance that require further investigation. This opens up the potential for these biomarkers to be used clinically to reduce the need for invasive procedures, aid in diagnosing and treating patients with chemoresistant GBM, and target specific lncRNAs associated with chemoresistance. LncRNAs have proven to be valuable for diagnostic and prognostic applications. Excessive experimental and clinical studies are necessary to identify the most sensitive and specific markers associated with high plasma levels in GBM subtypes and aggressiveness, and to explore the feasibility of using lncRNAs as a quick, easy, and noninvasive diagnostic and therapeutic approach for GBM. Incorporating lncRNAs into diagnostic and prognostic strategies may improve GBM patient care by enhancing precision medicine. The use of lncRNA-based biomarkers for both early detection and prognosis supports a dual-purpose model that can improve patient stratification and early diagnosis before clinical manifestations. Serving as reliable molecular indicators, lncRNAs show promise as non-invasive liquid biopsy tests that not only confirm GBM but also inform clinicians about disease progression (178).

Future research should move beyond identifying individual oncogenic or tumor-suppressive lncRNAs and focus on understanding how lncRNA networks coordinate immune evasion within the glioblastoma microenvironment. Targeting immunomodulatory lncRNAs may represent a complementary strategy to enhance immune recognition and overcome resistance to current immunotherapies. While lncRNAs hold great potential as therapeutic agents for GBM due to their ability to regulate oncogenic pathways and modulate the tumor microenvironment, their delivery in the brain faces numerous obstacles (203). Overcoming the BBB, ensuring targeted and efficient delivery, preventing off-target effects, and developing stable and effective delivery systems are key areas that require further research (231). 
